# Improvement in variance estimation using transformed auxiliary variable under simple random sampling

**DOI:** 10.1038/s41598-024-58841-x

**Published:** 2024-04-06

**Authors:** Hameed Ali, Syed Muhammad Asim, Muhammad Ijaz, Tolga Zaman, Soofia Iftikhar

**Affiliations:** 1 Department of Statistics, University of Peshawar, Peshawar, Pakistan; 2 Department of Mathematics and Statistics, University of Haripur, Harīpur, Pakistan; 3 Department of Statistics, Cankiri Karatekin University, Çankırı, Turkey; 4 Department of Statistics, Shaheed Benazir Bhutto Women University, Peshawar, Pakistan

**Keywords:** Auxiliary variable, Mean square error, Population variance, Percentage relative efficiency, Applied mathematics, Statistics

## Abstract

This paper offers a novel approach to formulate efficient ratio estimator of the population variance using a transformed auxiliary variable. The impact of transformation on auxiliary information has also been discussed. It is observed that incorporating a transformed auxiliary variable result in a high gain in efficiency. Theoretical properties of the newly developed estimators have been derived. The empirical and simulation studies show that the suggested estimators outperformed the existing estimators.

## Introduction

Sampling is a crucial aspect of making well-informed decisions in various real-life domains. Inferences about statistical populations or data are drawn from samples, and it is imperative that a sample accurately represents every characteristic of the population of interest. Data is characterized by specific parameters, and estimating population parameters from a sample poses a challenging task. Two essential measures for specifying data are the measure of location and scale. This article focuses on the latter, specifically the estimation of variance, and a frequently used measure of data scale. Estimating variance is vital in processes where precise quantification of data variation is necessary. From economics and business to physical science, biological science, and environmental sciences, sophisticated tools are required to measure variation for making informed decisions. For instance, economists analyze the variation in commodity prices, manufacturers assess taste preferences for customer satisfaction, and agriculturists study variability in climate factors to optimize yields and minimize costs.

Extensive research has sought to enhance the efficiency of ratio and product estimators for finite population variance, considering the correlation between survey variables and auxiliary variables. Auxiliary variables are those correlated with the main study variable, either positively or negatively. In environmental studies, for example, auxiliary variables like wind speed or temperature are considered when estimating air quality variance. Economic surveys may involve estimating household income variance using employment rates as an auxiliary variable. In healthcare planning, patient demographics or medical histories serve as auxiliary variables when estimating variance in patient recovery times.

Integrating auxiliary variables with the main survey variable to estimate variance provides additional information, refining the accuracy of estimates. This additional information includes contextual data, environmental factors, or supplementary metrics correlated with main study variable. The use of auxiliary information often leads to more efficient and robust variance estimators. Similarly, the transformative role is notable in enhancing estimate efficacy. Transformations make estimators flexible, allowing them to better utilize additional information from auxiliary variables, such as mean, variance, skewness, kurtosis, quantiles, etc. This flexibility, along with generalization and optimization constants, enhances the robustness of estimates against variations in the sample.

That is why various sample survey statisticians preferred transformed auxiliary variables instead of considering them in their original form. Keeping in view the above, numerous researchers developed many efficient estimators of population variance. Some of the pioneer work on estimating variance of finite population using auxiliary variable are due to^1^ who proposed an unbiased estimator of variance and^2^ compares variance estimators under various sample designs with available auxiliary information. Illustrates improved bias and mean squared error over common estimators. Similarly^3^, introduces chain estimators for finite population variance using double sampling and two auxiliary variables. Compares estimators based on mean square estimator (MSE) criterion. Proposes a ratio-type exponential estimator for population variance, consistently more efficient than previous estimators^4^. Conducts efficiency comparisons mathematically and numerically. Introduces a family of estimators based on adaptations of previous work, showing efficiency through mean square error comparisons^5^. A generalized modified ratio-type estimator for population variance using known parameters of the auxiliary variable was suggested by^6^. Compares with existing estimators for simulated and real data to show the performance of the developed estimator against the competing estimators. Suggests a generalized class of finite population variance, deriving large sample bias and mean square error. Considers special cases and provides numerical examples for comparison^7^. Suggested estimator of population variance utilizing information on two auxiliary variables under SRSWOR scheme^8^. A generalized exponential estimator for estimating population variance using two auxiliary variables was proposed by^9^. Demonstrates efficiency through empirical and simulated studies using real and simulated data. Has suggested efficient formulation of population variance in simple random sampling using supplementary variable^10^. Using searl’s constants, develop an efficient estimator to estimate the population variance^11^. The bias and mean squared error of the proposed estimator is obtained up to the first degree of approximation. Suggested a chain ratio type and chain ratio type exponential estimator of variance of finite population using auxiliary information Improved version of the suggested class of estimators is also given along with its properties^12^. An empirical study is carried out in support of the findings of the study. Addresses estimation of current population variance in the presence of random non-response^13^. Examines proposed estimators through empirical studies, comparing with estimators for complete response situations. Suggested a class of estimators for finite population variance using an auxiliary attribute^14^. Developed variance estimator using the tri-mean and third quartile of the auxiliary variable, demonstrating its superior performance over various competing estimators based on sampling properties, bias, and mean squared error^15^. Suggested finite population variance estimator using unconventional measures^16^. Demonstrates efficacy and robustness through empirical and simulation studies considering real data sets form various domain of life. Similarly,^17^ explores the effect of distribution on suggested variance estimators. Compares twelve estimators across eight distributions through simulation studies. Formulate a Searl’s ratio-type estimator using tri-mean and third quartile of the auxiliary variable^18^. Demonstrates superiority through bias, mean squared error through theoretical comparison and empirical studies. A hybrid-type estimators of population variance developed by^19^, and demonstrated the efficiency over competing estimators through theoretical and empirical comparisons. A generalized family of estimators of population variance is formulated by^20^ and demonstrated the performance of the estimators through empirical and simulation study. A robust ratio-type estimator for finite population variance suggested by^21^, considering robust covariance matrices. Derive conditions for efficiency against competing estimators and demonstrated the performance of the suggested estimators through empirical and simulation study.

## Novelty and significance

This work introduces innovative contributions in the field of survey sampling, with several key aspects. The paper puts forward three novel ratio estimators designed for finite population variance. These estimators consider a transformed auxiliary variable under simple random sampling without replacement. Demonstrated to outperform existing methods, these suggested estimators exhibit superior efficacy when there is a positive correlation between the survey variable and auxiliary variable. In addition to theoretical comparisons of mean squared errors (MSEs), the paper includes an empirical analysis using real data sets. Through a simulation study, it substantiates that the newly proposed estimators consistently outperform competing estimators across various scenarios, such as different correlation and sample size, affirming their high efficiency. The versatility of the proposed estimators is highlighted as they can seamlessly adapted into other sampling methodologies. This includes applications in stratified random sampling, non-response sampling, and adaptive cluster sampling, leading to the derivation of efficient versions of the estimators. The applicability of the proposed estimators extends to diverse fields such as environmental studies, agriculture, and economics, especially in situations where a positive correlation between the study and auxiliary variable exists. The heightened efficiency of these estimators can significantly enhance the accuracy of population variance estimates, offering practical implications for decision-making processes in real-world applications. The proposed estimator can significantly contribute in the estimation of parameters other than variance, such as mean, median, coefficient of variation etc.

In conclusion, this paper significantly contributes to the field of survey sampling by introducing novel estimators for finite population variance. The adaptability of these estimators to various sampling schemes and their practical implications underscores their potential impact on enhancing accuracy in real-world decision-making.

## Methodology

Consider a population $\Omega = \left\{ {\left( {y_{i} ,x_{i} } \right)} \right\},i = 1,2,...N.$ of size N. Suppose a random sample $\left( {y_{i} ,z_{i} } \right)$ of size n is taken from a population under simple random sampling without replacement case, i-e (SRSWOR). Let $\left( {y_{i} ,x_{i} } \right)$ be the value of *i*th unit of the main study and auxiliary variable and $z_{i} = t\left( {x_{i} } \right)$ is the transformed auxiliary variable observed on the sample. The supplementary variate $\left( x \right)$ is supposed to be correlated positively with the main study variable $\left( y \right)$. It is to be noted that the correlation between $\left( {y_{i} ,z_{i} } \right)$ and between $\left( {y_{i} ,x_{i} } \right)$ is same. Let $\overline{y} = \frac{1}{n}\sum\limits_{i = 1}^{n} {y_{i}^{{}} }$, $\overline{x} = \frac{1}{n}\sum\limits_{i = 1}^{n} {x_{i}^{{}} } \,and\,\,\overline{z} = \frac{1}{n}\sum\limits_{i = 1}^{n} {z_{i}^{{}} }$ represents the sample mean of study variable, ancillary variable and transformed ancillary variable.

$\overline{Y} = \frac{1}{N}\sum\limits_{i = 1}^{N} {y_{i}^{{}} }$ and $\overline{X} = \frac{1}{N}\sum\limits_{i = 1}^{N} {x_{i}^{{}} } \,\,and\,\,\,\overline{Z} = \frac{1}{N}\sum\limits_{i = 1}^{N} {z_{i}^{{}} }$ be the population mean of variate $\left( y \right)$,$\left( x \right)$ and $\left( z \right)$.

Let us define the random error due to sampling by $\varepsilon_{0} = \frac{{s_{y}^{2} - S_{y}^{2} }}{{S_{y}^{2} }}$ and $\varepsilon_{1} = \frac{{s_{x}^{2} - S_{x}^{2} }}{{S_{x}^{2} }}$, such that

1 $$ \left. \begin{gathered} {\rm E}\left( {\varepsilon_{0} } \right) = {\rm E}\left( {\varepsilon_{1} } \right) = 0\,and \hfill \\ {\rm E}\left( {\varepsilon_{0}^{2} } \right) = \lambda \left( {\phi_{40} - 1} \right) = V_{40} \hfill \\ \,{\rm E}\left( {\varepsilon_{1}^{2} } \right) = \lambda \left( {\phi_{04} - 1} \right) = V_{04} \, \hfill \\ \,\,{\rm E}\left( {\varepsilon_{0}^{{}} \varepsilon_{1}^{{}} } \right) = \lambda \left( {\phi_{22} - 1} \right) = V_{22} \hfill \\ where \hfill \\ \lambda = 1/n\,\,,\,\,\phi_{rs} = \frac{{\mu_{rs} }}{{\mu_{20}^{r/2} \mu_{02}^{s/2} }}\, \hfill \\ \mu_{rs} = \frac{1}{N}\sum\limits_{i = 1}^{N} {(y_{i} - \overline{Y})^{r} } (x_{i} - \overline{X})^{s} \hfill \\ \end{gathered} \right\}. $$

where *r and s* be the non-negative integer and $\mu_{rs} ,\mu_{20}^{{}} \;\;and\,\;\mu_{02}^{{}}$ are the second order moments and $\phi_{rs}$ is the moment’s ratio.

2 $$ \left. \begin{gathered} S_{y}^{2} = \frac{1}{N - 1}\sum\limits_{i = 1}^{N} {\left( {Y_{i} - \overline{Y}} \right)^{2} } ,s_{y}^{2} = \frac{1}{n - 1}\sum\limits_{i = 1}^{n} {(y_{i} - \overline{y})^{2} } \hfill \\ S_{x}^{2} = \frac{1}{N - 1}\sum\limits_{i = 1}^{N} {\left( {X_{i} - \overline{X}} \right)^{2} } ,S_{y}^{2} = \frac{1}{N - 1}\sum\limits_{i = 1}^{N} {(x_{i} - \overline{x})^{2} } \hfill \\ \end{gathered} \right\}. $$

where $C_{y}^{2} ,C_{x}^{2}$ are the coefficient of variation of the survey variable and auxiliary variables *Y* and *X* respectively.$\rho_{yx}$ is the correlation coefficient between main study variable Y and auxiliary variable X, and $\beta_{(1)x}^{{}} ,\beta_{(2)x}^{{}}$ are the coefficient of skewness and the coefficient of kurtosis of the auxiliary variables respectively.

In literature, some estimators of the population variance are given as

1. The usual classical estimator of population variance is given by
  3 $$ \tau_{1}^{{}} = s_{y}^{2} = \frac{1}{n - 1}\sum\limits_{i = 1}^{n} {\left( {y_{i} - \overline{y}} \right)^{2} } , $$
  where $\tau_{1}^{{}}$ is an unbiased estimator. Its variance is as under
  4 $$ Var\left( {\tau_{1} } \right) = S_{y}^{4} V_{40} . $$
2. Developed the estimator of population variance, it is given by^2^
  5 $$ \tau_{2}^{{}} = s_{y}^{2} \left( {\frac{{S_{x}^{2} }}{{s_{x}^{2} }}} \right), $$

The MSE of $\tau_{2}$ is as below.

6 $$ MSE\left( {\tau_{2} } \right) \cong S_{y}^{4} \left\{ {V_{40} + V_{04} - 2V_{22} } \right\} $$

3. Provide the estimator of population variance, it is given below^22^
  7 $$ \tau_{3}^{{}} = s_{y}^{2} \exp \left( {\frac{{S_{x}^{2} - s_{x}^{2} }}{{S_{x}^{2} + s_{x}^{2} }}} \right), $$

The MSE of $\tau_{3}^{{}}$ is given by

8 $$ MSE\left( {\tau_{3}^{{}} } \right) \approx S_{y}^{4} \left\{ {V_{40} + \frac{{V_{04} }}{4} - V_{22} } \right\}. $$

4. For ratio estimator of population variance, the linear regression estimator developed by^2^ is given by
  9 $$ \tau_{4}^{{}} = s_{y}^{2} + b\left( {S_{x}^{2} - s_{x}^{2} } \right), $$
  here $b = \frac{{s_{y}^{2} V_{22} }}{{s_{x}^{2} V_{40} }}$ represents the sample regression coefficient.

The variance of $\tau_{4}^{{}}$ is as below

10 $$ Var\left( {\tau_{4}^{{}} } \right) = S_{y}^{4} V_{40} \left( {1 - \frac{{V_{22}^{2} }}{{V_{40} V_{04} }}} \right). $$

5. The transformed estimator by^23^ is as follows:
  11 $$ \tau_{5}^{{}} = \left\{ {k_{1} s_{y}^{2} + k_{2} \left( {S_{x}^{2} - s_{x}^{2} } \right)} \right\}\left\{ {\theta \left( {\frac{{aS_{x}^{2} + b}}{{as_{x}^{2} + b}}} \right) + \left( {1 - \theta } \right)\exp \left( {\frac{{a\left( {S_{x}^{2} - s_{x}^{2} } \right)}}{{a\left( {S_{x}^{2} + s_{x}^{2} } \right) + 2b}}} \right)} \right\}, $$
  here $k_{1}$ and $k_{2}$ are optimization constants, $\theta$ is generalization constants which can takes value between 0 and 1 and “a” and “b” some function of auxiliary variable. The optimum value of $k_{1}$ and $k_{1}$ are as below
  $$ k_{{1\left( {opt} \right)}} = \left( {\frac{{1 - \tfrac{1}{8}q^{2} \left( {1 + 3\theta + 4\theta^{2} } \right)V_{04} }}{{1 - \tfrac{1}{4}q^{2} \theta \left( {1 + 3\theta } \right)V_{04}^{2} + V_{40} \left( {1 - \frac{{V_{22}^{2} }}{{V_{04} V_{40} }}} \right)}}} \right) $$
  and
  $$ k_{{2\left( {opt} \right)}} = \frac{{S_{y}^{2} }}{{S_{x}^{2} }}\left\{ {1 + q\left( {1 + \theta } \right) + k_{{1\left( {opt} \right)}} \left( {\frac{{V_{22} }}{{V_{04} }} - q\left( {1 + \theta } \right)} \right)} \right\}, $$

The minimum MSE at $k_{{1\left( {opt} \right)}}$ and $k_{{2\left( {opt} \right)}}$ is given by

12 $$ MSE\left( {\tau_{5} } \right) \simeq S_{y}^{4} \left[ {\left\{ {1 - \frac{1}{4}q^{2} \left( {1 + \theta } \right)^{2} V_{04} } \right\} - \frac{{\left\{ {1 - \tfrac{1}{8}q^{2} \left( {1 + 3\theta + 4\theta^{2} } \right)V_{04} } \right\}^{2} }}{{1 - \frac{1}{4}q^{2} \theta \left( {1 + 3\theta } \right)V_{04}^{2} + V_{40} \left( {1 - \frac{{V_{22}^{2} }}{{V_{04} V_{40} }}} \right)}}} \right]. $$

where $$q = \frac{{aS_{x}^{2} }}{{aS_{x}^{2} + b}}.$$

The MSE of $\tau$ is minimum at $\left( {\theta ,a,b} \right) = \left( {1,1,0} \right)$.

6. Introduced the following difference-cum-exponential estimator of population variance^7^,
  13 $$ \tau_{6} = \left[ {c_{11} s_{y}^{2} + c_{22} \left( {S_{x}^{2} - s_{x}^{2} } \right)} \right]\exp \left( {\frac{{S_{x}^{2} - s_{x}^{2} }}{{S_{x}^{2} + s_{x}^{2} }}} \right), $$
  where $c_{11}$ and $c_{11}$ are optimization constants. The optimum MSE of $\tau_{6}$ is given by
  14 $$ MSE\left( {\tau_{6} } \right) = MSE\left( {\tau_{4} } \right) - \frac{{S_{y}^{4} \left[ {\left( {V_{40}^{{}} - 1} \right) + 8V_{04}^{{}} \left( {1 - \frac{{V_{22}^{2} }}{{V_{40}^{{}} V_{04}^{{}} }}} \right)} \right]}}{{64\left\{ {1 + \left\{ {V_{40}^{{}} - \frac{{V_{22}^{2} }}{{V_{04}^{{}} }}} \right\}} \right\}}}. $$
7. Developed the following ratio estimator of population variance^24^
  15 $$ \tau_{7}^{{}} = s_{y}^{2} \left[ {k_{11} \left( {\frac{{S_{x}^{2} }}{{s_{x}^{2} }}} \right) + k_{12} \left( {\frac{{s_{x}^{2} }}{{S_{x}^{2} }}} \right)} \right]\exp \left( {\frac{{S_{x}^{2} - s_{x}^{2} }}{{S_{x}^{2} + s_{x}^{2} }}} \right) $$

The optimum value of $k_{11} \,\,and\,\,k_{12}$ are given by

$$    k_{{11_{{OPT}} }}  =  - \frac{1}{8}\frac{{A_{1} V_{{40}}  + V_{{04}}  + A_{2}  + 16V_{{22}} }}{{A_{3} }}  $$

$$  k_{{12_{{OPT}} }}  = \frac{1}{8}\frac{{A_{1} V_{{40}}  + 21V_{{04}}  - 3A_{2}  + 16V_{{22}} }}{{A_{3} }}, $$

where

$$ \begin{gathered} A_{1} = 16\left( {V_{04} - V_{22} } \right), \hfill \\ A_{2} = 8\left( {3V_{04} V_{22} - 2V_{22}^{2} + V_{04} } \right) \hfill \\ and \hfill \\ A_{3} = V_{04}^{2} - 4V_{04} V_{22} - 16V_{04} V_{22} + 16V_{22}^{2} - 4V_{04} . \hfill \\ \end{gathered} $$

The minimum MSE at $k_{{11\left( {opt} \right)}} \,\,and\,\,k_{{12\left( {opt} \right)}}$ is given by

16 $$ MSE\left( {\tau_{7}^{{}} } \right)_{\min } \cong \,\frac{{S_{y}^{4} }}{{A_{3}^{{}} }}\left[ {\frac{1}{16}\left\{ \begin{subarray}{l} 16V_{22}^{{}} \left\{ {V_{22}^{{}} \left( {4V_{40}^{{}} - 3V_{04}^{{}} + 4} \right) + \left( {3V_{04}^{2} - 8V_{40}^{{}} V_{04}^{{}} } \right)} \right\} \\ \,\,\,\,\,\,\,\,\,\,\,\,\,\,\,\,\,\,\,\,\,\,\,\,\,\,\,\,\,\,\,\,\,\,\,\,\,\,\,\, + 9V_{04}^{3} + 64V_{04}^{{}} \left( {V_{04}^{{}} - 1} \right)V_{40}^{{}} \end{subarray} \right\}} \right]. $$

8. Advocated the following difference-cum-exponential type exponential estimators of population variance given by^25^
  17 $$ \tau_{8} = \left[ {\frac{{s_{y}^{2} }}{2}\left\{ {\exp \left( {\frac{{S_{x}^{2} - s_{x}^{2} }}{{S_{x}^{2} + s_{x}^{2} }}} \right) + \exp \left( {\frac{{s_{x}^{2} - S_{x}^{2} }}{{S_{x}^{2} + s_{x}^{2} }}} \right)} \right\} + k_{1}^{{}} \left( {S_{x}^{2} - s_{x}^{2} } \right) + k_{2}^{{}} s_{y}^{2} } \right]\exp \left( {\frac{{S_{x}^{2} - s_{x}^{2} }}{{S_{x}^{2} + s_{x}^{2} }}} \right), $$
  18 $$ \begin{gathered} \tau_{9} = \left[ \begin{gathered} s_{y}^{2} \left\{ {\alpha \exp \left( {\frac{{S_{x}^{2} - s_{x}^{2} }}{{S_{x}^{2} + s_{x}^{2} }}} \right) + \left( {1 - \alpha } \right)\exp \left( {\frac{{s_{x}^{2} - S_{x}^{2} }}{{S_{x}^{2} + s_{x}^{2} }}} \right)} \right\} \hfill \\ \,\,\,\,\,\,\,\,\,\,\,\,\,\,\,\,\,\,\,\,\,\,\,\,\,\,\,\,\,\,\,\,\,\,\,\,\,\,\,\,\,\,\,\, + k_{11}^{{}} \left( {S_{x}^{2} - s_{x}^{2} } \right) + k_{22}^{{}} s_{y}^{2} \hfill \\ \end{gathered} \right]\left\{ \begin{gathered} \delta \exp \left( {\frac{{S_{x}^{2} - s_{x}^{2} }}{{S_{x}^{2} + s_{x}^{2} }}} \right) \hfill \\ + \left( {1 - \delta } \right)\exp \left( {\frac{{s_{x}^{2} - S_{x}^{2} }}{{S_{x}^{2} + s_{x}^{2} }}} \right) \hfill \\ \end{gathered} \right\} \hfill \\ \,\,\,\,\,\,\,\,\,\,\,\,\,\,\,\,\,\,\,\,\,\,\,\,\,\,\,\,\,\,\,\,\,\,\,\,\,\,\,\,\,\,\,\,\,\,\,, \hfill \\ \end{gathered} $$
  where $k_{1}^{{}}$ and $k_{2}^{{}}$ are optimization constants that minimize the MSE of $\tau_{8}$.which is given by
  19 $$  MSE\left( {\tau _{8} } \right) \cong MSE\left( {\tau _{4} } \right) - \frac{{S_{y}^{4} \left\{ {\left( {V_{{40}}^{{}}  - \frac{{V_{{22}} ^{2} }}{{V_{{04}}^{{}} }}} \right) + \frac{1}{4}V_{{04}} ^{2} } \right\}}}{{\left\{ {1 + \left( {V_{{40}}^{{}}  - \frac{{V_{{22}} ^{2} }}{{V_{{04}}^{{}} }}} \right)} \right\}}}. $$
  20 $$ MSE\left( {\tau_{9} } \right) \cong S_{y}^{4} \left[ {\left\{ {V_{40}^{{}} + \Omega^{{}} \left( {\Omega V_{04}^{{}} + 2V_{22}^{{}} } \right)} \right\} - \frac{{D_{1}^{{}} D_{5}^{2} + D_{2}^{{}} D_{4}^{2} - 2D_{3}^{{}} D_{4}^{{}} D_{5}^{{}} }}{{D_{1}^{{}} D_{2}^{{}} - D_{3}^{2} }}} \right]. $$
  $$ \begin{gathered} D_{1} = R^{2} V_{04} , \hfill \\ D_{2} = 1 + \left\{ {V_{04} + 2V_{22} \left( {1 - 2\omega } \right) + \omega^{2} V_{04} } \right\}, \hfill \\ D_{3} = R\left[ {V_{22} + \left( {1 - 2\omega } \right)V_{04} } \right], \hfill \\ D_{4} = R\left[ {V_{22} + \Omega V_{04} } \right] \hfill \\ \end{gathered} $$
  and
  $$ D_{5} = \left[ {V_{40} + \left\{ {\left\{ {2\Omega + \frac{1 - 2\omega }{2}} \right\}V_{22} + \left\{ {\alpha \omega + \frac{1 - 2\omega }{2}} \right\}V_{04} } \right\}} \right]. $$
9. Suggested a general type of estimator of population variance given by^20^
  21 $$ \tau_{{\left( {a,b} \right)}}^{{}} = \left[ {\left( {\hat{t}_{{\left( {a,b} \right)}} + k\left( {S_{x}^{2} - s_{x}^{2} } \right)} \right)\exp \left( {\frac{{w_{1} \left( {\overline{X} - \overline{x}} \right)}}{{\overline{X} + \left( {\omega_{1} - 1} \right)\overline{x}}} + \frac{{w_{2} \left( {S_{x}^{2} - s_{x}^{2} } \right)}}{{S_{x}^{2} + \left( {\omega_{2} - 1} \right)s_{x}^{2} }}} \right)} \right], $$

The particular case of estimator $\tau_{9}^{a,b}$ for estimating variance is obtained by putting a = 0, b = 2,$w_{1} = 0\,and\,\,w_{2} = 1$ and $\omega_{2}$ =  2 as following

22 $$ \tau_{9}^{{}} = \left[ {\left( {s_{y}^{2} + k\left( {S_{x}^{2} - s_{x}^{2} } \right)} \right)\exp \left( {\frac{{\left( {S_{x}^{2} - s_{x}^{2} } \right)}}{{S_{x}^{2} + s_{x}^{2} }}} \right)} \right], $$

With MSE given by

23 $$ \begin{gathered} MSE\left( t \right)_{\min } = MSE\left( {\hat{t}_{(a,b)} } \right) \hfill \\ - \frac{{t^{2}_{(a,b)} }}{n}\frac{{\left[ {\left\{ {f_{3} \left( {a,b} \right)} \right\}^{2} - 2f_{2} \left( {a,b} \right)f_{3} \left( {a,b} \right)\delta_{03} + \left( {\delta_{04} - 1} \right)\left\{ {f_{2} \left( {a,b} \right)} \right\}^{2} } \right]}}{{\left( {\delta_{04} - \delta_{03}^{2} - 1} \right)}} \hfill \\ \end{gathered} $$

where

$$ \begin{gathered} f_{2} \left( {a,b} \right) = \left\{ {a\rho_{XY} C_{\gamma } + \left( \frac{b}{2} \right)\delta_{21} } \right\} \hfill \\ f_{3} \left( {a,b} \right) = \left\{ {a\rho_{12} C_{\gamma } + \left( \frac{b}{2} \right)\left( {\delta_{22} - 1} \right)} \right\}. \hfill \\ \end{gathered} $$

Which in case of variance estimator, the MSE $\tau$ will induce the following particular case:

24 $$ MSE\left( {\tau_{9}^{{}} } \right) = MSE\left( {\tau_{1} } \right) - \frac{{\left[ {\left\{ {f_{3} \left( {0,2} \right)} \right\}^{2} - 2f_{2} \left( {0,2} \right)V_{40} + \left( {V_{04} - 1} \right)\left\{ {f_{2} \left( {0,2} \right)} \right\}^{2} } \right]}}{{\left( {V_{04} - V_{40}^{2} - 1} \right)}} $$

## Proposed estimators

### The first proposed estimator

Motivated by^24^ and using transformed auxiliary variable, the following class of transformed ratio-product type exponential estimator is suggestedor

$$ \tau_{P1}^{{}} = s_{y}^{2} \left[ {c_{1} \left( \frac{Z}{z} \right) + c_{2} \left( \frac{z}{Z} \right)} \right]\exp \left( {\frac{Z - z}{{Z + z}}} \right) $$

25 $$ \tau_{P1}^{{}} = s_{y}^{2} \left[ {c_{1} \left( {\frac{{\gamma_{1} S_{x}^{2} - \gamma_{2} }}{{\gamma_{1} s_{x}^{2} - \gamma_{2} }}} \right) + c_{2} \left( {\frac{{\gamma_{1} s_{x}^{2} - \gamma_{2} }}{{\gamma_{1} S_{x}^{2} - \gamma_{2} }}} \right)} \right]\exp \left( {\frac{{\gamma_{1} \left( {S_{x}^{2} - s_{x}^{2} } \right)}}{{\gamma_{1} \left( {S_{x}^{2} + s_{x}^{2} } \right) - 2\gamma_{2} }}} \right) $$

where $c_{1}$ and $c_{2}$ are optimization constants and $\gamma_{2}$ and $\gamma_{2}$ are suitable constants or some function of auxiliary variables.

### The second proposed estimator

Motivated by^2^, we can write the proposed estimator as a linear combination of usual ratio and exponential estimators as followingOr

$$ \tau_{P2}^{{}} = \psi_{1} \tau_{2}^{{}} + \psi_{2} \tau_{3}^{{}} $$

26 $$ \tau_{P2}^{{}} = s_{y}^{2} \left\{ {\psi_{1} \left( {\frac{{S_{x}^{2} }}{{s_{x}^{2} }}} \right) + \psi_{2} \exp \left( {\frac{{S_{x}^{2} - s_{x}^{2} }}{{S_{x}^{2} + s_{x}^{2} }}} \right)} \right\} $$

$\psi_{1}$, and $\psi_{2}$ are optimization constants whose value is to be obtained so that the MSE of $\tau_{P2}^{{}}$ is minimum.

### The third proposed estimator

Applying transformation to the auxiliary variable in (26), we can write the third proposed estimator as following

27 $$ \tau_{P3}^{{}} = s_{y}^{2} \left\{ {\psi_{3} \left( \frac{Z}{z} \right) + \psi_{4} \exp \left( {\frac{Z - z}{{Z + z}}} \right)} \right\} $$

Or

$$ \tau_{P3}^{{}} = s_{x}^{2} \left\{ {\psi_{3} \left( {\frac{{\gamma_{1} S_{x}^{2} - \gamma_{2} }}{{\gamma_{1} s_{x}^{2} - \gamma_{2} }}} \right) + \psi_{4} \exp \left( {\frac{{\gamma_{1} \left( {S_{x}^{2} - s_{x}^{2} } \right)}}{{\gamma_{1} \left( {S_{x}^{2} + s_{x}^{2} } \right) - 2\gamma_{2} }}} \right)} \right\} $$

$\psi_{3} \,$ and $\psi_{4} \,$ are optimization constants whose value is to be determined so that the MSE of $\tau_{P3}^{{}}$ is minimum. It is to be noted that for $\gamma_{1} = 1\,\,and\,\,\gamma_{2} = 0$.

The third proposed estimator $\tau_{P3}^{{}}$ given by (26) become equivalent to the second proposed estimator $\tau_{P2}^{{}}$ given by (26).

Similarly, the first proposed estimator $\tau_{P1}^{{}}$ given by (25) become equivalent to $\tau_{P7}^{{}}$ as suggested by Muneer et al.^9^ given by (13).

## Theoretical properties of the proposed estimators

This unit aims at, deriving the theoretical properties of the new estimators using the notations given in (1) and (2). Rewriting (25), (26) and (27) respectively in term of error terms, as following

$$ \tau_{P1}^{{}} = S_{y}^{2} \left( {1 + \varepsilon_{0} } \right)\left[ {c_{1} \left( {1 - \pi_{k} \varepsilon_{1} + \pi_{k}^{2} \varepsilon_{1}^{2} + \cdots } \right) + c_{2} \left( {1 + \pi_{k} \varepsilon_{1} } \right)} \right]\left( {1 - \frac{1}{2}\pi_{k} \varepsilon_{1} + \frac{3}{8}\pi_{k}^{2} \varepsilon_{1}^{2} + \cdots } \right), $$

$$ \tau_{P2}^{{}} = S_{y}^{2} \left( {1 + \varepsilon_{0} } \right)\left\{ {\psi_{1} \left( {1 - \varepsilon_{1} + \varepsilon_{1}^{2} + \cdots } \right) + \psi_{2} \left( {1 - \frac{1}{2}\varepsilon_{1}^{{}} + \frac{3}{8}\varepsilon_{1}^{2} + \cdots } \right)} \right\}, $$

and

$$ \tau_{P3}^{{}} = S_{y}^{2} \left( {1 + \varepsilon_{0} } \right)\left\{ {\psi_{3} \left( {1 - \pi_{k}^{{}} \varepsilon_{1} + \pi_{k}^{2} \varepsilon_{1}^{2} + \cdots } \right) + \psi_{4} \left( {1 - \frac{1}{2}\pi_{k}^{{}} \varepsilon_{1}^{{}} + \frac{3}{8}\pi_{k}^{2} \varepsilon_{1}^{2} + \cdots } \right)} \right\}, $$

where $$\pi_{k} = \frac{{\gamma_{1} S_{x}^{2} }}{{\gamma_{1} S_{x}^{2} - \gamma_{2} }}\,\,\,.$$ or

28 $$ \tau_{P1}^{{}} - S_{y}^{2} \cong S_{y}^{2} \left[ {c_{1} \left( {1 + \varepsilon_{0} - \pi_{k} \varepsilon_{1} - \frac{3}{2}\pi_{k} \varepsilon_{1} \varepsilon_{0} + \frac{15}{8}\pi_{k}^{2} \varepsilon_{1}^{2} } \right) + c_{2} \left( \begin{gathered} 1 + \varepsilon_{0} + \frac{1}{2}\pi_{k} \varepsilon_{1} + \hfill \\ \frac{1}{2}\pi_{k} \varepsilon_{1} \varepsilon_{0} - \frac{1}{8}\pi_{k}^{2} \varepsilon_{1}^{2} \hfill \\ \end{gathered} \right) - 1} \right] $$

29 $$ \tau_{P2}^{{}} - S_{y}^{2} \cong S_{y}^{2} \left[ \begin{gathered} \left( {\psi_{1} + \psi_{2} - 1} \right) + \left( {\psi_{1} + \psi_{2} } \right)\varepsilon_{0} - \left( {\psi_{1} + \frac{{\psi_{2} }}{2}} \right)\varepsilon_{1} + \left( {\psi_{1} + \frac{{3\psi_{2} }}{8}} \right)\varepsilon_{1}^{2} \hfill \\ \,\,\,\,\,\,\,\,\,\,\,\,\,\,\,\,\,\,\,\,\,\,\,\,\,\,\,\,\,\,\,\,\,\,\,\,\,\,\,\,\,\,\,\,\,\,\,\,\,\,\,\,\,\,\,\,\,\,\,\,\,\,\,\,\,\,\,\,\,\,\,\,\,\,\,\,\,\,\,\,\,\,\,\,\,\,\,\,\, - \left( {\psi_{1} + \frac{{\psi_{2} }}{2}} \right)\varepsilon_{0} \varepsilon_{1} \hfill \\ \end{gathered} \right], $$

and

30 $$ \tau_{P3}^{{}} - S_{y}^{2} \cong S_{y}^{2} \left[ \begin{gathered} \left( {\psi_{3} + \psi_{4} - 1} \right) + \left( {\psi_{3} + \psi_{4} } \right)\varepsilon_{0} - \pi_{k} \left( {\psi_{3} + \frac{{\psi_{4} }}{2}} \right)\varepsilon_{1} + \left( {\psi_{3} + \frac{{3\psi_{4} }}{8}} \right)\pi_{k}^{2} \varepsilon_{1}^{2} \hfill \\ \,\,\,\,\,\,\,\,\,\,\,\,\,\,\,\,\,\,\,\,\,\,\,\,\,\,\,\,\,\,\,\,\,\,\,\,\,\,\,\,\,\,\,\,\,\,\,\,\,\,\,\,\,\,\,\,\,\,\,\,\,\,\,\,\,\,\,\,\,\,\,\,\,\,\,\,\,\,\,\,\,\,\,\, - \pi_{k} \left( {\psi_{4} + \frac{{\psi_{4} }}{2}} \right)\varepsilon_{0} \varepsilon_{1} \hfill \\ \end{gathered} \right], $$

Taking expectation of both sides of (28), (29) and (30) respectively, and after simplification we get

31 $$ Bias\left( {\tau_{P1}^{{}} } \right) \cong S_{y}^{2} \left[ \begin{gathered} \left( {c_{1} + c_{2} - 1} \right) + c_{1} \lambda \left\{ {\frac{15}{8}\pi_{k}^{2} \left( {\phi_{04}^{{}} - 1} \right) - \frac{1}{2}\pi_{k} \left( {\phi_{02}^{{}} - 1} \right)} \right\} \hfill \\ \,\,\,\,\,\,\,\,\,\,\,\,\,\,\,\,\,\,\,c_{1} \lambda \pi_{k} \left\{ {\frac{1}{2}\pi_{k} \left( {\phi_{02}^{{}} - 1} \right) - \frac{1}{8}\pi_{k}^{{}} \left( {\phi_{04}^{{}} - 1} \right)} \right\} \hfill \\ \end{gathered} \right], $$

32 $$ Bias\left( {\tau_{P2}^{{}} } \right) \cong S_{y}^{2} \left[ {\left( {\psi_{1} + \psi_{2} - 1} \right) + \left( {\psi_{1} + \frac{{3\psi_{2} }}{8}} \right)\lambda \left( {\phi_{04}^{{}} - 1} \right) - \lambda \left( {\psi_{1} + \frac{{\psi_{2} }}{2}} \right)\left( {\phi_{22}^{{}} - 1} \right)} \right], $$

33 $$ Bias\left( {\tau_{P3}^{{}} } \right) \cong S_{y}^{2} \left[ {\left( {\psi_{3} + \psi_{4} - 1} \right) + \left( {\psi_{3} + \frac{{3\psi_{4} }}{8}} \right)\pi_{k}^{2} \lambda \left( {\phi_{04}^{{}} - 1} \right) - \pi_{k} \lambda \left( {\psi_{3} + \frac{{\psi_{4} }}{2}} \right)\left( {\phi_{22}^{{}} - 1} \right)} \right], $$

Squaring both sides of (31), (32) and (33) respectively and applying expectation to get the MSE of $\tau_{P1}^{{}} ,\tau_{P2}^{{}} \,\,and\,\,\tau_{P3}^{{}}$, as following.

34 $$ {\rm E}\left( {\tau_{P1}^{{}} - S_{y}^{2} } \right)^{2} \cong S_{y}^{4} {\rm E}\left[ \begin{gathered} c_{1} \left( {1 + \varepsilon_{0} - \pi_{k} \varepsilon_{1} - \frac{3}{2}\pi_{k} \varepsilon_{1} \varepsilon_{0} + \frac{15}{8}\pi_{k}^{2} \varepsilon_{1}^{2} } \right) + \hfill \\ c_{2} \left( {1 + \varepsilon_{0} + \frac{1}{2}\pi_{k} \varepsilon_{1} + \frac{1}{2}\pi_{k} \varepsilon_{1} \varepsilon_{0} - \frac{1}{8}\pi_{k}^{2} \varepsilon_{1}^{2} } \right) - 1 \hfill \\ \end{gathered} \right]^{2} , $$

35 $$ E\left( {\tau_{P2}^{{}} - S_{y}^{2} } \right) \cong S_{y}^{4} E\left[ \begin{gathered} \left( {\psi_{1} + \psi_{2} - 1} \right) + \left( {\psi_{1} + \psi_{2} } \right)\varepsilon_{0} - \left( {\psi_{1} + \frac{{\psi_{2} }}{2}} \right)\varepsilon_{1} + \hfill \\ \left( {\psi_{1} + \frac{{3\psi_{2} }}{8}} \right)\varepsilon_{1}^{2} - \left( {\psi_{1} + \frac{{\psi_{2} }}{2}} \right)\varepsilon_{0} \varepsilon_{1} \hfill \\ \end{gathered} \right]^{2} , $$

or

36 $$ E\left( {\tau_{P3}^{{}} - S_{y}^{2} } \right) \cong S_{y}^{4} E\left[ \begin{gathered} \left( {\psi_{3} + \psi_{4} - 1} \right) + \left( {\psi_{3} + \psi_{4} } \right)\varepsilon_{0} - \pi_{k} \left( {\psi_{3} + \frac{{\psi_{4} }}{2}} \right)\varepsilon_{1} + \hfill \\ \left( {\psi_{3} + \frac{{3\psi_{4} }}{8}} \right)\pi_{k}^{2} \varepsilon_{1}^{2} - \pi_{k} \left( {\psi_{3} + \frac{{\psi_{4} }}{2}} \right)\varepsilon_{0} \varepsilon_{1} \hfill \\ \end{gathered} \right]^{2} , $$

or

37 $$ MSE\left( {\tau_{P1}^{{}} } \right) \cong S_{y}^{4} \left[ \begin{gathered} \left( {c_{1} + c_{2} - 1} \right)^{2} + \left( {c_{1} + c_{2} } \right)^{2} V_{40} + \left\{ \begin{gathered} \left( {\frac{1}{2}\left( {3c_{1} - c_{2} } \right)} \right)^{2} + \hfill \\ 2\left( {c_{1} + c_{2} - 1} \right)\left( {\frac{15}{8}c_{1} - \frac{1}{8}c_{2} } \right) \hfill \\ \end{gathered} \right\}\pi_{k}^{2} V_{04} \hfill \\ \,\,\,\,\,\,\,\,\,\,\,\,\,\,\,\,\,\,\,\,\,\,\,\,\,\,\,\,\,\,\,\,\,\,\,\,\,\,\,\,\,\,\,\,\,\,\,\,\,\,\,\,\,\,\,\,\,\,\,\,\, - \left( {2\left( {c_{1} + c_{2} } \right) - 1} \right)\left( {3c_{1} - c_{2} } \right)\pi_{k} V_{22} \hfill \\ \end{gathered} \right], $$

38 $$ MSE\left( {\tau_{P2}^{{}} } \right) \cong S_{y}^{4} \left[ \begin{gathered} \left( {\psi_{1} + \psi_{2} - 1} \right)^{2} + \left( {\psi_{1} + \psi_{2} } \right)^{2} V_{40} + \left\{ \begin{gathered} \left( {\psi_{1} + \frac{{\psi_{2} }}{2}} \right)^{2} + \hfill \\ 2\left( {\psi_{1} + \psi_{2} - 1} \right)\left( {\psi_{1} + \frac{{3\psi_{2} }}{8}} \right) \hfill \\ \end{gathered} \right\}V_{04} \hfill \\ - 2\left( {\psi_{1} + \frac{{\psi_{2} }}{2}} \right)\left\{ {2\left( {\psi_{1} + \psi_{2} } \right) - 1} \right\}V_{22} \hfill \\ \end{gathered} \right] $$

and

39 $$ MSE\left( {\tau_{P3}^{{}} } \right) \cong S_{y}^{4} \left[ \begin{gathered} \left( {\psi_{3} + \psi_{4} - 1} \right)^{2} + \left( {\psi_{3} + \psi_{4} } \right)^{2} V_{40} + \left\{ \begin{gathered} \left( {\psi_{3} + \frac{{\psi_{4} }}{2}} \right)^{2} + \hfill \\ 2\left( {\psi_{3} + \psi_{4} - 1} \right)\left( {\psi_{3} + \frac{{3\psi_{4} }}{8}} \right) \hfill \\ \end{gathered} \right\}\pi_{k}^{2} V_{04} \hfill \\ \,\,\,\,\,\,\,\,\,\,\,\,\,\,\,\,\,\,\,\,\,\,\,\,\,\,\,\,\,\,\,\,\,\,\,\,\,\,\,\,\,\,\,\,\,\,\,\,\,\,\,\,\,\,\,\,\,\,\,\,\,\,\, - 2\left( {\psi_{3} + \frac{{\psi_{4} }}{2}} \right)\left\{ {2\left( {\psi_{3} + \psi_{4} } \right) - 1} \right\}\pi_{k} V_{22} \hfill \\ \end{gathered} \right]. $$

The optimum value of $c_{1}$ and $c_{2}$ is obtain by differentiating (37) w.r.t $c_{1} \,and\,\,c_{2}$ respectively and equating to zero, as following

$$  \frac{\partial }{{\partial c_{1} }}{\text{MSE}}\left( {\tau _{{P1}}^{{}} } \right){\text{ = 0}} \Rightarrow \,c_{{1_{{OPT}} }}  =  - \frac{1}{8}\frac{{A_{{1,k}} V_{{40}}  + V_{{04,k}}  + A_{{2,k}}  + 16V_{{22,k}} }}{{A_{{3,k}} }},  $$

$$  \frac{\partial }{{\partial c_{2} }}{\text{MSE}}\left( {\tau _{{P1}}^{{}} } \right){\text{ = 0}} \Rightarrow c_{{2_{{OPT}} }}  = \frac{1}{8}\frac{{A_{{1,k}} V_{{40}}  + 21V_{{04,k}}  - 3A_{{2,k}}  + 16V_{{22,k}} }}{{A_{{3,k}} }}.  $$

where,

$$ \begin{gathered} A_{1,k} = 16\left( {V_{04,k} - V_{22,k} } \right), \hfill \\ A_{2,k} = 8\left( {3V_{04,k} V_{22,k} - 2V_{22,k}^{2} + V_{04,k} } \right), \hfill \\ and \hfill \\ A_{3,k} = V_{04,k}^{2} - 4V_{04,k} V_{22,k} - 16V_{04,k} V_{22,k} + 16V_{22,k}^{2} - 4V_{04,k} . \hfill \\ \end{gathered} $$

putting the optimum value of $c_{1} \,\,and\,\,c_{2}$ in (37), we get

40 $$_{{MSE\left( {\tau_{P1}^{{}} } \right)_{\min } \cong \frac{{S_{y}^{4} }}{{A_{3,k}^{{}} }}\left[ {\frac{1}{16}\left\{ \begin{subarray}{l} 16V_{22,k}^{{}} \left\{ {V_{22,k}^{{}} \left( {4V_{40}^{{}} - 3V_{04,k}^{{}} + 4} \right) + \left( {3V_{04,k}^{2} - 8V_{40}^{{}} V_{04,k}^{{}} } \right)} \right\} + \\ \,\,\,\,\,\,\,\,\,\,\,\,\,\,9V_{04,k}^{3} + 64V_{04,k}^{{}} \left( {V_{04,k}^{{}} - 1} \right)V_{40}^{{}} \end{subarray} \right\}} \right].}} $$

where

$$ \,\,V_{04,k} = \pi_{k}^{2} V_{04} \,\,and\,\,V_{22,k} = \pi_{k}^{{}} V_{22} $$

Similarly, using calculus rule, the optimum value of $\psi_{1} ,\psi_{2} ,\psi_{3} \,\,and\,\psi_{4}$ can also be obtain by differentiating MSE of $\tau_{P2} \,\,and\,\,\tau_{P3}$ w.r.to $\psi_{1} ,\psi_{2} ,\psi_{3} \,\,and\,\psi_{4}$ and equating to zero. Hence, we obtain after simplification

$$ \psi_{{1\left( {opt} \right)}} = - \frac{{19V_{04}^{4} + 40V_{04}^{2} V_{40}^{2} - 60V_{04}^{2} V_{22}^{{}} - 32V_{40}^{2} V_{22}^{{}} + 32V_{22}^{2} - 16V_{04}^{2} + 32V_{22}^{{}} }}{D}, $$

$$ \psi_{{2\left( {opt} \right)}} = \frac{{8\left( {6V_{04}^{4} + 5V_{04}^{2} V_{40}^{2} - 15V_{04}^{2} V_{22}^{{}} - 4V_{40}^{2} V_{22}^{{}} + 8\lambda V_{22}^{2} - 4V_{04}^{2} + 4V_{22}^{{}} } \right)}}{D}, $$

$$ \psi_{{3\left( {opt} \right)}} = - \frac{{19V_{04,k}^{4} + 40V_{04,k}^{2} V_{40}^{2} - 60V_{04,k}^{2} V_{22,k}^{{}} - 32V_{40}^{2} V_{22,k}^{{}} + 32V_{22,k}^{2} - 16V_{04,k}^{2} + 32V_{22,k}^{{}} }}{{D_{k} }} $$

and

$$ \psi_{{4\left( {opt} \right)}} = \frac{{8\left( {6V_{04,k}^{4} + 5V_{04,k}^{2} V_{40}^{2} - 15V_{04,k}^{2} V_{22,k}^{{}} - 4V_{40}^{2} V_{22,k}^{{}} + 8\lambda V_{22,k}^{2} - 4V_{04,k}^{2} + 4V_{22,k}^{{}} } \right)}}{{D_{k} }} $$

where

$$ \begin{aligned} D & = 33V_{04}^{4} - 16V_{04}^{2} V_{40}^{2} - 80V_{04}^{2} V_{22}^{{}} + 64V_{22}^{2} - 16V_{04}^{2} \\ D_{k} & = 33V_{04,k}^{4} - 16V_{04,k}^{2} V_{40}^{2} - 80V_{04,k}^{2} V_{22,k}^{{}} + 64V_{22,k}^{2} - 16V_{04,k}^{2} ,\;\;k = 1,2, \ldots ,6. \\ \end{aligned} $$

The MSE is given by

41 $$ {\text{MSE}}\left( {t_{P2} } \right) = \frac{{S_{y}^{4} }}{D}\left( {\left( \begin{gathered} V_{04}^{6} + \left( {25V_{40}^{2} - 10V_{22}^{2} } \right)V_{04}^{4} + \hfill \\ V_{04}^{2} \left( {8V_{22}^{{}} - 40V_{22}^{{}} V_{40}^{2} } \right) + 16V_{22}^{2} V_{40}^{2} \hfill \\ \end{gathered} \right) - 16V_{04}^{2} V_{40}^{2} + 16V_{22}^{2} } \right) $$

42 $$ {\text{MSE}}\left( {\tau_{P3} } \right) = \frac{{S_{y}^{4} }}{{D_{k} }}\left( {\left( \begin{gathered} V_{04,k}^{6} + \left( {25V_{40}^{2} - 10V_{22,k}^{2} } \right)V_{04,k}^{4} + \hfill \\ V_{04,k}^{2} \left( {8V_{22,k}^{{}} - 40V_{22,k}^{{}} V_{40}^{2} } \right) + 16V_{22,k}^{2} V_{40}^{2} \hfill \\ \end{gathered} \right) - 16V_{04,k}^{2} V_{40}^{2} + 16V_{22,k}^{2} } \right) $$

### Special Cases of $\tau_{P1}^{{}} \,\,and\,\,\tau_{P3}^{{}}$ in response to the transformation introduced

1. For k = 1, $\tau_{P1}^{{}} \,\,and\,\,\tau_{P3}^{{}}$ takes the following form
  $$ \tau_{P1,1}^{{}} = s_{y}^{2} \left[ {c_{1} \left( {\frac{{S_{x}^{2} - \rho_{yx} }}{{s_{x}^{2} - \rho_{yx} }}} \right) + c_{2} \left( {\frac{{s_{x}^{2} - \rho_{yx} }}{{S_{x}^{2} - \rho_{yx} }}} \right)} \right]\exp \left( {\frac{{S_{x}^{2} - s_{x}^{2} }}{{S_{x}^{2} + s_{x}^{2} - 2\rho_{yx} }}} \right) $$
  $$ \tau_{P3,1}^{{}} = s_{y}^{2} \left\{ {\psi_{3} \left( {\frac{{S_{x}^{2} - \rho_{yx} }}{{s_{x}^{2} - \rho_{yx} }}} \right) + \psi_{4} \exp \left( {\frac{{S_{x}^{2} - s_{x}^{2} }}{{S_{x}^{2} + s_{x}^{2} - 2\rho_{yx} }}} \right)} \right\}. $$
  where $$\gamma_{1} = 1\,\,and\,\,\gamma_{2} = \rho_{yx} \,\,\,$$

The bias and MSEs are given by

43 $$ Bias\left( {\tau_{P1,1}^{{}} } \right) = S_{y\left( h \right)}^{2} \left[ {\left( {c_{1} + c_{2} - 1} \right) + \left( {\frac{3}{2}c_{1} - \frac{1}{2}c_{2} } \right)V_{22,1} + \,\left( {\frac{15}{8}c_{1} - \frac{1}{8}c_{2} } \right)V_{04,1} } \right], $$

44 $$ Bias\left( {\tau_{P3,1}^{{}} } \right) \cong S_{y}^{2} \left[ {\left( {\psi_{3} + \psi_{4} - 1} \right) + \left( {\psi_{3} + \frac{{3\psi_{4} }}{8}} \right)V_{04,1} - \left( {\psi_{1} + \frac{{\psi_{2} }}{2}} \right)V_{22,1} } \right] $$

45 $$_{{MSE\left( {\tau_{P1,1}^{{}} } \right)_{\min } \cong \frac{{S_{y}^{4} }}{{A_{3,1}^{{}} }}\left[ {\frac{1}{16}\left\{ \begin{subarray}{l} 16V_{22,1}^{{}} \left\{ {V_{22,1}^{{}} \left( {4V_{40}^{{}} - 3V_{04,1}^{{}} + 4} \right) + \left( {3V_{04,1}^{2} - 8V_{40}^{{}} V_{04,1}^{{}} } \right)} \right\} + \\ \,\,\,\,\,\,\,\,\,\,\,\,\,\,9V_{04,1}^{3} + 64V_{04,1}^{{}} \left( {V_{04,1}^{{}} - 1} \right)V_{40}^{{}} \end{subarray} \right\}} \right]}} $$

and

46 $$ {\text{MSE}}\left( {\tau_{P3,1} } \right) = \frac{{S_{y}^{4} }}{{D_{1} }}\left( {\left( \begin{gathered} V_{04,1}^{6} + \left( {25V_{40}^{2} - 10V_{22,1}^{2} } \right)V_{04,1}^{4} + \hfill \\ V_{04,1}^{2} \left( {8V_{22,1}^{{}} - 40V_{22,1}^{{}} V_{40}^{2} } \right) + 16V_{22,1}^{2} V_{40}^{2} \hfill \\ \end{gathered} \right) - 16V_{04,1}^{2} V_{40}^{2} + 16V_{22,1}^{2} } \right). $$

$$\,\,V_{04,1} = \pi_{1}^{2} V_{04} \,\,and\,\,V_{22,1} = \pi_{1}^{{}} V_{22}$$, $$\pi_{1}^{{}} = \frac{{S_{x}^{2} }}{{S_{x}^{2} - \rho_{yx} }}.$$

2. For k = 2, $\gamma_{1} = 1\,\,and\,\,\gamma_{2} = C_{x}$, $\tau_{P1}^{{}} \,\,and\,\,\tau_{P3}$ will take the following form
  $$ \tau_{P1,2}^{{}} = s_{y}^{2} \left[ {c_{1} \left( {\frac{{S_{x}^{2} - C_{x} }}{{s_{x}^{2} - C_{x} }}} \right) + c_{2} \left( {\frac{{s_{x}^{2} - C_{x} }}{{S_{x}^{2} - C_{x} }}} \right)} \right]\exp \left( {\frac{{S_{x}^{2} - s_{x}^{2} }}{{S_{x}^{2} + s_{x}^{2} - 2C_{x} }}} \right). $$
  $$ \tau_{P3,2}^{{}} = s_{y}^{2} \left\{ {\psi_{3} \left( {\frac{{S_{x}^{2} - C_{x} }}{{s_{x}^{2} - C_{x} }}} \right) + \psi_{4} \exp \left( {\frac{{S_{x}^{2} - s_{x}^{2} }}{{S_{x}^{2} + s_{x}^{2} - 2C_{x} }}} \right)} \right\}. $$

The bias and MSEs are given by

47 $$ Bias\left( {\tau_{P1,2}^{{}} } \right) = S_{y\left( h \right)}^{2} \left[ {\left( {c_{1} + c_{2} - 1} \right) + \left( {\frac{3}{2}c_{1} - \frac{1}{2}c_{2} } \right)V_{22,2} + \,\left( {\frac{15}{8}c_{1} - \frac{1}{8}c_{2} } \right)V_{04,2} } \right], $$

48 $$ Bias\left( {\tau_{P3,2}^{{}} } \right) \cong S_{y}^{2} \left[ {\left( {\psi_{3} + \psi_{4} - 1} \right) + \left( {\psi_{3} + \frac{{3\psi_{4} }}{8}} \right)V_{04,2} - \left( {\psi_{1} + \frac{{\psi_{2} }}{2}} \right)V_{22,2} } \right]. $$

49 $$_{{MSE\left( {\tau_{P1,2}^{{}} } \right)_{\min } \cong \frac{{S_{y}^{4} }}{{A_{3,2}^{{}} }}\left[ {\frac{1}{16}\left\{ \begin{subarray}{l} 16V_{22,2}^{{}} \left\{ {V_{22,2}^{{}} \left( {4V_{40}^{{}} - 3V_{04,2}^{{}} + 4} \right) + \left( {3V_{04,2}^{2} - 8V_{40}^{{}} V_{04,2}^{{}} } \right)} \right\} + \\ \,\,\,\,\,\,\,\,\,\,\,\,\,\,9V_{04,2}^{3} + 64V_{04,2}^{{}} \left( {V_{04,2}^{{}} - 1} \right)V_{40}^{{}} \end{subarray} \right\}} \right]}} $$

and

50 $$ {\text{MSE}}\left( {\tau_{P3,2} } \right) = \frac{{S_{y}^{4} }}{{D_{2} }}\left( {\left( \begin{gathered} V_{04,2}^{6} + \left( {25V_{40}^{2} - 10V_{22,2}^{2} } \right)V_{04,2}^{4} + \hfill \\ V_{04,2}^{2} \left( {8V_{22,2}^{{}} - 40V_{22,2}^{{}} V_{40}^{2} } \right) + 16V_{22,2}^{2} V_{40}^{2} \hfill \\ \end{gathered} \right) - 16V_{04,2}^{2} V_{40}^{2} + 16V_{22,2}^{2} } \right). $$

where $$\,\,V_{04,2} = \pi_{2}^{2} V_{04} \,\,and\,\,V_{22,2} = \pi_{2}^{{}} V_{22}$$,$$\pi_{2}^{{}} = \frac{{S_{x}^{2} }}{{S_{x}^{2} - C_{x} }}.$$

3. For k = 3, $\,\gamma_{1} = \rho_{yx} \,\,and\,\,\gamma_{2} = C_{x}$,$\tau_{P1}^{{}} \,\,and\,\,\tau_{P3}$ will take the following form
  $$ \tau_{P1,3}^{{}} = s_{y}^{2} \left[ {c_{1} \left( {\frac{{\rho_{yx} S_{x}^{2} - C_{x} }}{{\rho_{yx} s_{x}^{2} - C_{x} }}} \right) + c_{2} \left( {\frac{{\rho_{yx} s_{x}^{2} - C_{x} }}{{\rho_{yx} S_{x}^{2} - C_{x} }}} \right)} \right]\exp \left( {\frac{{\rho_{yx} \left( {S_{x}^{2} - s_{x}^{2} } \right)}}{{\rho_{yx} \left( {S_{x}^{2} + s_{x}^{2} } \right) - 2C_{x} }}} \right) $$
  $$ \tau_{P3,3}^{{}} = s_{y}^{2} \left\{ {\psi_{3} \left( {\frac{{\rho_{yx} S_{x}^{2} - C_{x} }}{{\rho_{yx} s_{x}^{2} - C_{x} }}} \right) + \psi_{4} \exp \left( {\frac{{\rho_{yx} \left( {S_{x}^{2} - s_{x}^{2} } \right)}}{{\rho_{yx} \left( {S_{x}^{2} + s_{x}^{2} } \right) - 2C_{x} }}} \right)} \right\}. $$

The bias along with the mean square error (MSE) is given by

51 $$ Bias\left( {\tau_{P1,3}^{{}} } \right) = S_{y}^{2} \left[ {\left( {c_{1} + c_{2} - 1} \right) + \left( {\frac{3}{2}c_{1} - \frac{1}{2}c_{2} } \right)V_{22,3} + \,\left( {\frac{15}{8}c_{1} - \frac{1}{8}c_{2} } \right)V_{04,3} } \right], $$

52 $$ Bias\left( {\tau_{P3,3}^{{}} } \right) \cong S_{y}^{2} \left[ {\left( {\psi_{3} + \psi_{4} - 1} \right) + \left( {\psi_{3} + \frac{{3\psi_{4} }}{8}} \right)V_{04,3} - \left( {\psi_{1} + \frac{{\psi_{2} }}{2}} \right)V_{22,3} } \right] $$

53 $$_{{MSE\left( {\tau_{P1,3}^{{}} } \right)_{\min } \cong \frac{{S_{y}^{4} }}{{A_{3,3}^{{}} }}\left[ {\frac{1}{16}\left\{ \begin{subarray}{l} 16V_{22,3}^{{}} \left\{ {V_{22,3}^{{}} \left( {4V_{40}^{{}} - 3V_{04,3}^{{}} + 4} \right) + \left( {3V_{04,3}^{2} - 8V_{40}^{{}} V_{04,3}^{{}} } \right)} \right\} + \\ \,\,\,\,\,\,\,\,\,\,\,\,\,\,9V_{04,3}^{3} + 64V_{04,3}^{{}} \left( {V_{04,3}^{{}} - 1} \right)V_{40}^{{}} \end{subarray} \right\}} \right]}} $$

and

54 $$ {\text{MSE}}\left( {\tau_{P3,3} } \right) = \frac{{S_{y}^{4} }}{{D_{3} }}\left( {\left( \begin{gathered} V_{04,3}^{6} + \left( {25V_{40}^{2} - 10V_{22,3}^{2} } \right)V_{04,3}^{4} + \hfill \\ V_{04,3}^{2} \left( {8V_{22,3}^{{}} - 40V_{22,3}^{{}} V_{40}^{2} } \right) + 16V_{22,3}^{2} V_{40}^{2} \hfill \\ \end{gathered} \right) - 16V_{04,3}^{2} V_{40}^{2} + 16V_{22,3}^{2} } \right). $$

where $$\,\,V_{04,3} = \pi_{3}^{2} V_{04} \,\,and\,\,V_{22,3} = \pi_{3}^{{}} V_{22}$$,$$\pi_{3}^{{}} = \frac{{\rho_{yx} S_{x}^{2} }}{{\rho_{yx} S_{x}^{2} - C_{x} }}.$$

4. For k = 4, $\,\gamma_{1} = C_{x} \,\,and\,\,\gamma_{2} = \rho_{yx} .$ the estimators $\tau_{P1}^{{}} \,\,and\,\,\tau_{P3}$ will take the following form
  $$ \begin{gathered} \tau_{P1,4}^{{}} = s_{y}^{2} \left[ {c_{1} \left( {\frac{{C_{x} S_{x}^{2} - \rho_{yx} }}{{C_{x} s_{x}^{2} - \rho_{yx} }}} \right) + c_{2} \left( {\frac{{C_{x} s_{x}^{2} - \rho_{yx} }}{{C_{x} S_{x}^{2} - \rho_{yx} }}} \right)} \right]\exp \left( {\frac{{C_{x} \left( {S_{x}^{2} - s_{x}^{2} } \right)}}{{C_{x} \left( {S_{x}^{2} + s_{x}^{2} } \right) - 2\rho_{yx} }}} \right) \hfill \\ \,\,\,\,\,\,\,\,\,\,\,\,\,\,\,\,\,\,\,\,\tau_{P3,4}^{{}} = s_{y}^{2} \left\{ {\psi_{3} \left( {\frac{{C_{x} S_{x}^{2} - \rho_{yx} }}{{C_{x} s_{x}^{2} - \rho_{yx} }}} \right) + \psi_{4} \exp \left( {\frac{{C_{x} \left( {S_{x}^{2} - s_{x}^{2} } \right)}}{{C_{x} \left( {S_{x}^{2} + s_{x}^{2} } \right) - 2\rho_{yx} }}} \right)} \right\}. \hfill \\ \end{gathered} $$

The bias and MSEs are obtained as

55 $$ Bias\left( {\tau_{P1,4}^{{}} } \right) = S_{y}^{2} \left[ {\left( {c_{1} + c_{2} - 1} \right) + \left( {\frac{3}{2}c_{1} - \frac{1}{2}c_{2} } \right)V_{22,4} + \,\left( {\frac{15}{8}c_{1} - \frac{1}{8}c_{2} } \right)V_{04,4} } \right], $$

56 $$ Bias\left( {\tau_{P3,4}^{{}} } \right) \cong S_{y}^{2} \left[ {\left( {\psi_{3} + \psi_{4} - 1} \right) + \left( {\psi_{3} + \frac{{3\psi_{4} }}{8}} \right)V_{04,4} - \left( {\psi_{1} + \frac{{\psi_{2} }}{2}} \right)V_{22,4} } \right] $$

57 $$_{{MSE\left( {\tau_{P1,4}^{{}} } \right)_{\min } \cong \frac{{S_{y}^{4} }}{{A_{3,4}^{{}} }}\left[ {\frac{1}{16}\left\{ \begin{subarray}{l} 16V_{22,4}^{{}} \left\{ {V_{22,4}^{{}} \left( {4V_{40}^{{}} - 3V_{04,4}^{{}} + 4} \right) + \left( {3V_{04,4}^{2} - 8V_{40}^{{}} V_{04,4}^{{}} } \right)} \right\} + \\ \,\,\,\,\,\,\,\,\,\,\,\,\,\,9V_{04,4}^{3} + 64V_{04,4}^{{}} \left( {V_{04,4}^{{}} - 1} \right)V_{40}^{{}} \end{subarray} \right\}} \right]}} $$

and

58 $$ {\text{MSE}}\left( {\tau_{P3,4} } \right) = \frac{{S_{y}^{4} }}{{D_{4} }}\left( {\left( \begin{gathered} V_{04,4}^{6} + \left( {25V_{40}^{2} - 10V_{22,4}^{2} } \right)V_{04,4}^{4} + \hfill \\ V_{04,4}^{2} \left( {8V_{22,4}^{{}} - 40V_{22,4}^{{}} V_{40}^{2} } \right) + 16V_{22,4}^{2} V_{40}^{2} \hfill \\ \end{gathered} \right) - 16V_{04,4}^{2} V_{40}^{2} + 16V_{22,4}^{2} } \right). $$

where $$\,\,V_{04,1} = \pi_{1}^{2} V_{04} \,\,and\,\,V_{22,1} = \pi_{1}^{{}} V_{22}$$,$$\pi_{4}^{{}} = \frac{{C_{x} S_{x}^{2} }}{{C_{x} S_{x}^{2} - \rho_{yx} }}.$$

5. For k = 5, $\gamma_{1} = \overline{X}\,and\,\,\gamma_{2} = \rho_{yx} \,$, the estimators $\tau_{P1}^{{}} \,\,and\,\,\tau_{P3}$ will take the following form
  $$ \begin{gathered} \tau_{P1,5}^{{}} = s_{y}^{2} \left[ {c_{1} \left( {\frac{{\overline{X}S_{x}^{2} - \rho_{yx} }}{{\overline{X}s_{x}^{2} - \rho_{yx} }}} \right) + c_{2} \left( {\frac{{\overline{X}s_{x}^{2} - \rho_{yx} }}{{\overline{X}S_{x}^{2} - \rho_{yx} }}} \right)} \right]\exp \left( {\frac{{\overline{X}\left( {S_{x}^{2} - s_{x}^{2} } \right)}}{{\overline{X}\left( {S_{x}^{2} + s_{x}^{2} } \right) - 2\rho_{yx} }}} \right) \hfill \\ \tau_{P3,5}^{{}} = s_{y}^{2} \left\{ {\psi_{3} \left( {\frac{{\overline{X}S_{x}^{2} - \rho_{yx} }}{{\overline{X}s_{x}^{2} - \rho_{yx} }}} \right) + \psi_{4} \exp \left( {\frac{{\overline{X}\left( {S_{x}^{2} - s_{x}^{2} } \right)}}{{\overline{X}\left( {S_{x}^{2} + s_{x}^{2} } \right) - 2\rho_{yx} }}} \right)} \right\}. \hfill \\ \end{gathered} $$

The bias and MSEs are given by

59 $$ Bias\left( {\tau_{P1,5}^{{}} } \right) = S_{y}^{2} \left[ {\left( {c_{1} + c_{2} - 1} \right) + \left( {\frac{3}{2}c_{1} - \frac{1}{2}c_{2} } \right)V_{22,5} + \,\left( {\frac{15}{8}c_{1} - \frac{1}{8}c_{2} } \right)V_{04,5} } \right], $$

60 $$ Bias\left( {\tau_{P3,5}^{{}} } \right) \cong S_{y}^{2} \left[ {\left( {\psi_{3} + \psi_{4} - 1} \right) + \left( {\psi_{3} + \frac{{3\psi_{4} }}{8}} \right)V_{04,5} - \left( {\psi_{1} + \frac{{\psi_{2} }}{2}} \right)V_{22,5} } \right] $$

61 $$_{{MSE\left( {\tau_{P1,5}^{{}} } \right)_{\min } \cong \frac{{S_{y}^{4} }}{{A_{3,5}^{{}} }}\left[ {\frac{1}{16}\left\{ \begin{subarray}{l} 16V_{22,5}^{{}} \left\{ {V_{22,5}^{{}} \left( {4V_{40}^{{}} - 3V_{04,5}^{{}} + 4} \right) + \left( {3V_{04,5}^{2} - 8V_{40}^{{}} V_{04,5}^{{}} } \right)} \right\} + \\ \,\,\,\,\,\,\,\,\,\,\,\,\,\,9V_{04,5}^{3} + 64V_{04,5}^{{}} \left( {V_{04,5}^{{}} - 1} \right)V_{40}^{{}} \end{subarray} \right\}} \right]}} $$

and

62 $$ {\text{MSE}}\left( {\tau_{P3,5} } \right) = \frac{{S_{y}^{4} }}{{D_{5} }}\left( {\left( \begin{gathered} V_{04,5}^{6} + \left( {25V_{40}^{2} - 10V_{22,5}^{2} } \right)V_{04,5}^{4} + \hfill \\ V_{04,5}^{2} \left( {8V_{22,5}^{{}} - 40V_{22,5}^{{}} V_{40}^{2} } \right) + 16V_{22,5}^{2} V_{40}^{2} \hfill \\ \end{gathered} \right) - 16V_{04,5}^{2} V_{40}^{2} + 16V_{22,5}^{2} } \right). $$

where $$\,\,V_{04,5} = \pi_{5}^{2} V_{04} \,\,and\,\,V_{22,5} = \pi_{5}^{{}} V_{22}$$ and $$\pi_{5}^{{}} = \frac{{\overline{X}S_{x}^{2} }}{{\overline{X}S_{x}^{2} - \rho_{yx} }}.$$

6. For k = 6, $$\gamma_{1} = \rho_{yx} \,\,and\,\,\gamma_{2} = 1$$

$$ \begin{gathered} \tau_{P1,6}^{{}} = s_{y}^{2} \left[ {c_{1} \left( {\frac{{\rho_{yx} S_{x}^{2} - 1}}{{\rho_{yx} s_{x}^{2} - 1}}} \right) + c_{2} \left( {\frac{{\rho_{yx} s_{x}^{2} - 1}}{{\rho_{yx} S_{x}^{2} - 1}}} \right)} \right]\exp \left( {\frac{{\rho_{yx} \left( {S_{x}^{2} - s_{x}^{2} } \right)}}{{\rho_{yx} \left( {S_{x}^{2} + s_{x}^{2} } \right) - 2}}} \right) \hfill \\ \tau_{P3,6}^{{}} = s_{y}^{2} \left\{ {\psi_{3} \left( {\frac{{\rho_{yx} S_{x}^{2} - 1}}{{\rho_{yx} s_{x}^{2} - 1}}} \right) + \psi_{4} \exp \left( {\frac{{\rho_{yx} \left( {S_{x}^{2} - s_{x}^{2} } \right)}}{{\rho_{yx} \left( {S_{x}^{2} + s_{x}^{2} } \right) - 2}}} \right)} \right\}. \hfill \\ \end{gathered} $$

The bias and MSEs are given by

63 $$ Bias\left( {\tau_{P1,6}^{{}} } \right) = S_{y}^{2} \left[ {\left( {c_{1} + c_{2} - 1} \right) + \left( {\frac{3}{2}c_{1} - \frac{1}{2}c_{2} } \right)V_{22,6} + \,\left( {\frac{15}{8}c_{1} - \frac{1}{8}c_{2} } \right)V_{04,6} } \right], $$

64 $$ Bias\left( {\tau_{P3,6}^{{}} } \right) \cong S_{y}^{2} \left[ {\left( {\psi_{3} + \psi_{4} - 1} \right) + \left( {\psi_{3} + \frac{{3\psi_{4} }}{8}} \right)V_{04,6} - \left( {\psi_{1} + \frac{{\psi_{2} }}{2}} \right)V_{22,6} } \right] $$

65 $$_{{MSE\left( {\tau_{P1,6}^{{}} } \right)_{\min } \cong \frac{{S_{y}^{4} }}{{A_{3,6}^{{}} }}\left[ {\frac{1}{16}\left\{ \begin{subarray}{l} 16V_{22,6}^{{}} \left\{ {V_{22,6}^{{}} \left( {4V_{40}^{{}} - 3V_{04,6}^{{}} + 4} \right) + \left( {3V_{04,6}^{2} - 8V_{40}^{{}} V_{04,6}^{{}} } \right)} \right\} + \\ \,\,\,\,\,\,\,\,\,\,\,\,\,\,9V_{04,6}^{3} + 64V_{04,6}^{{}} \left( {V_{04,6}^{{}} - 1} \right)V_{40}^{{}} \end{subarray} \right\}} \right]}} $$

and

66 $$ {\text{MSE}}\left( {\tau_{P3,6} } \right) = \frac{{S_{y}^{4} }}{{D_{6} }}\left( {\left( \begin{gathered} V_{04,6}^{6} + \left( {25V_{40}^{2} - 10V_{22,6}^{2} } \right)V_{04,6}^{4} + \hfill \\ V_{04,6}^{2} \left( {8V_{22,6}^{{}} - 40V_{22,6}^{{}} V_{40}^{2} } \right) + 16V_{22,6}^{2} V_{40}^{2} \hfill \\ \end{gathered} \right) - 16V_{04,6}^{2} V_{40}^{2} + 16V_{22,6}^{2} } \right). $$

where $\,\,V_{04,6} = \pi_{6}^{2} V_{04} \,\,and\,\,V_{22,6} = \pi_{6}^{{}} V_{22w}$ and $\pi_{6\left( h \right)}^{{}} = \frac{{\rho_{\left( h \right)} S_{{w_{x} \left( h \right)}}^{2} }}{{\rho_{\left( h \right)} S_{{w_{x} \left( h \right)}}^{2} - 1}}.$ where $\,\,V_{04,6} = \pi_{6}^{2} V_{04} \,\,and\,\,V_{22,6} = \pi_{6}^{{}} V_{22}$, $\pi_{6}^{{}} = \frac{{S_{x}^{2} }}{{S_{x}^{2} - 1}}$.

### Efficiency comparisons

This section aims to compare the MSEs of the newly developed estimators with the competing estimators discussed in the literature.

**Condition 1.** By using (37), (38), (39) and (4) we can write

$$ MSE\left( {\tau_{Pi} } \right) - Var\left( {\tau_{1} } \right) < 0,\;\;\;{\text{i}}\, = \,{1},{2},{3}. $$

$$ {\text{For i}}\, = \,{1}\frac{{S_{y}^{4} }}{{A_{3,k}^{{}} }}\left[ {\frac{1}{16}\left\{ \begin{subarray}{l} 16V_{22,k}^{{}} \left\{ {V_{22,k}^{{}} \left( {4V_{40}^{{}} - 3V_{04,k}^{{}} + 4} \right) + \left( {3V_{04,k}^{2} - 8V_{40}^{{}} V_{04,k}^{{}} } \right)} \right\} + \\ \,\,\,\,\,\,\,\,\,\,\,\,\,\,9V_{04,k}^{3} + 64V_{04,k}^{{}} \left( {V_{04,k}^{{}} - 1} \right)V_{40}^{{}} - 16A_{3,k}^{{}} V_{40} \end{subarray} \right\}} \right] < 0 $$

$$ {\text{For i}}\, = \,{2}\frac{{S_{y}^{4} }}{D}\left( {\left( \begin{gathered} V_{04}^{6} + \left( {25V_{40}^{2} - 10V_{22}^{2} } \right)V_{04}^{4} + \hfill \\ V_{04}^{2} \left( {8V_{22}^{{}} - 40V_{22}^{{}} V_{40}^{2} } \right) + 16V_{22}^{2} V_{40}^{2} \hfill \\ \end{gathered} \right) - 16V_{04}^{2} V_{40}^{2} + 16V_{22}^{2} - DV_{40}^{{}} } \right) < 0. $$

$$ {\text{For i}}\, = \,{3}\frac{{S_{y}^{4} }}{{D_{k} }}\left( {\left( \begin{gathered} V_{04,k}^{6} + \left( {25V_{40}^{2} - 10V_{22,k}^{2} } \right)V_{04,k}^{4} + \hfill \\ V_{04,k}^{2} \left( {8V_{22,k}^{{}} - 40V_{22,k}^{{}} V_{40}^{2} } \right) + 16V_{22,k}^{2} V_{40}^{2} \hfill \\ \end{gathered} \right) - 16V_{04,k}^{2} V_{40}^{2} + 16V_{22,k}^{2} - D_{k} V_{40} } \right). $$

**Condition 2.** By using (37), (38), (39) and (6) we can write

$$MSE\left( {\tau_{Pi} } \right) - MSE\left( {\tau_{2} } \right) < 0,\;\;{\text{i}}\, = \,{1},{2},{3}.$$

For i = 1 $$\frac{{S_{y}^{4} }}{{A_{3,k}^{{}} }}\left[ {\frac{1}{16}\left\{ \begin{subarray}{l} 16V_{22,k}^{{}} \left\{ {V_{22,k}^{{}} \left( {4V_{40}^{{}} - 3V_{04,k}^{{}} + 4} \right) + \left( {3V_{04,k}^{2} - 8V_{40}^{{}} V_{04,k}^{{}} } \right)} \right\} + \\ \,\,\,\,\,\,\,\,\,\,\,\,\,\,9V_{04,k}^{3} + 64V_{04,k}^{{}} \left( {V_{04,k}^{{}} - 1} \right)V_{40}^{{}} - 16A_{3,k}^{{}} \left( {V_{40} + V_{04} - 2V_{22} } \right) \end{subarray} \right\}} \right] < 0$$.

For i = 2 $$\frac{{S_{y}^{4} }}{D}\left( \begin{gathered} \left( {V_{04}^{6} + \left( {25V_{40}^{2} - 10V_{22}^{2} } \right)V_{04}^{4} + V_{04}^{2} \left( {8V_{22}^{{}} - 40V_{22}^{{}} V_{40}^{2} } \right) + 16V_{22}^{2} V_{40}^{2} } \right) - \hfill \\ 16V_{04}^{2} V_{40}^{2} + 16V_{22}^{2} - D\left( {V_{40} + V_{04} - 2V_{22} } \right) \hfill \\ \end{gathered} \right) < 0$$.

For i = 3 $$\frac{{S_{y}^{4} }}{{D_{k} }}\left( \begin{gathered} \left( \begin{gathered} V_{04,k}^{6} + \left( {25V_{40}^{2} - 10V_{22,k}^{2} } \right)V_{04,k}^{4} + V_{04,k}^{2} \left( {8V_{22,k}^{{}} - 40V_{22,k}^{{}} V_{40}^{2} } \right) \hfill \\ + 16V_{22,k}^{2} V_{40}^{2} \hfill \\ \end{gathered} \right) \hfill \\ \,\,\,\,\,\,\,\,\,\,\,\,\,\,\,\,\,\,\,\,\,\,\,\,\,\,\,\,\,\,\,\,\,\,\, - 16V_{04,k}^{2} V_{40}^{2} + 16V_{22,k}^{2} - D_{k} \left( {V_{40} + V_{04} - 2V_{22} } \right) \hfill \\ \end{gathered} \right) < 0.$$

**Condition 3.** By using (37), (38), (39) and (8) we can write.

$$MSE\left( {\tau_{Pi} } \right) - MSE\left( {\tau_{3} } \right) < 0$$, i = 1,2,3.

For i = 1 $$\frac{{S_{y}^{4} }}{{A_{3,k}^{{}} }}\left[ {\frac{1}{16}\left\{ \begin{subarray}{l} 16V_{22,k}^{{}} \left\{ {V_{22,k}^{{}} \left( {4V_{40}^{{}} - 3V_{04,k}^{{}} + 4} \right) + \left( {3V_{04,k}^{2} - 8V_{40}^{{}} V_{04,k}^{{}} } \right)} \right\} + \\ \,\,\,\,\,\,\,\,\,\,\,\,\,\,9V_{04,k}^{3} + 64V_{04,k}^{{}} \left( {V_{04,k}^{{}} - 1} \right)V_{40}^{{}} - 16A_{3,k}^{{}} \left( {V_{40} + \frac{{V_{04} }}{4} - V_{22} } \right) \end{subarray} \right\}} \right] < 0$$.

For i = 2 $$\frac{{S_{y}^{4} }}{D}\left( \begin{gathered} \left( {V_{04}^{6} + \left( {25V_{40}^{2} - 10V_{22}^{2} } \right)V_{04}^{4} + V_{04}^{2} \left( {8V_{22}^{{}} - 40V_{22}^{{}} V_{40}^{2} } \right) + 16V_{22}^{2} V_{40}^{2} } \right) \hfill \\ \,\,\,\,\,\,\,\,\,\,\,\,\,\,\,\,\,\,\,\,\,\,\,\,\,\,\,\,\,\,\,\,\,\,\,\,\,\,\,\,\,\,\,\,\,\,\,\,\, - 16V_{04}^{2} V_{40}^{2} + 16V_{22}^{2} - D\left( {V_{40} + \frac{{V_{04} }}{4} - V_{22} } \right) \hfill \\ \end{gathered} \right) < 0$$.

For i = 3 $$\frac{{S_{y}^{4} }}{{D_{k} }}\left( \begin{gathered} \left( \begin{gathered} V_{04,k}^{6} + \left( {25V_{40}^{2} - 10V_{22,k}^{2} } \right)V_{04,k}^{4} + V_{04,k}^{2} \left( {8V_{22,k}^{{}} - 40V_{22,k}^{{}} V_{40}^{2} } \right) \hfill \\ + 16V_{22,k}^{2} V_{40}^{2} \hfill \\ \end{gathered} \right) \hfill \\ \,\,\,\,\,\,\,\,\,\,\,\,\,\,\,\,\,\,\,\,\,\,\,\,\,\,\,\,\,\,\,\,\,\,\, - 16V_{04,k}^{2} V_{40}^{2} + 16V_{22,k}^{2} - D_{k} \left( {V_{40} + \frac{{V_{04} }}{4} - V_{22} } \right) \hfill \\ \end{gathered} \right) < 0.$$

**Condition 4.** By using (37), (38), (39) and (10) we can write.

$$MSE\left( {\tau_{Pi} } \right) - MSE\left( {\tau_{4} } \right) < 0$$, i = 1,2,3.

For i = 1 $$\frac{{S_{y}^{4} }}{{A_{3,k}^{{}} }}\left[ {\frac{1}{16}\left\{ \begin{subarray}{l} 16V_{22,k}^{{}} \left\{ {V_{22,k}^{{}} \left( {4V_{40}^{{}} - 3V_{04,k}^{{}} + 4} \right) + \left( {3V_{04,k}^{2} - 8V_{40}^{{}} V_{04,k}^{{}} } \right)} \right\} + \\ \,\,\,\,\,\,\,\,\,\,\,\,\,\,9V_{04,k}^{3} + 64V_{04,k}^{{}} \left( {V_{04,k}^{{}} - 1} \right)V_{40}^{{}} - 16A_{3,k}^{{}} V_{40} \left( {1 - \frac{{V_{22}^{2} }}{{V_{40} V_{04} }}} \right) \end{subarray} \right\}} \right] < 0$$.

For i = 2 $$\frac{{S_{y}^{4} }}{D}\left( \begin{gathered} \left( {V_{04}^{6} + \left( {25V_{40}^{2} - 10V_{22}^{2} } \right)V_{04}^{4} + V_{04}^{2} \left( {8V_{22}^{{}} - 40V_{22}^{{}} V_{40}^{2} } \right) + 16V_{22}^{2} V_{40}^{2} } \right) \hfill \\ \,\,\,\,\,\,\,\,\,\,\,\,\,\,\,\,\,\,\,\,\,\,\,\,\,\,\,\,\,\,\,\,\,\,\,\,\,\,\,\,\,\,\,\,\,\,\,\,\, - 16V_{04}^{2} V_{40}^{2} + 16V_{22}^{2} - DV_{40} \left( {1 - \frac{{V_{22}^{2} }}{{V_{40} V_{04} }}} \right) \hfill \\ \end{gathered} \right) < 0$$.

For i = 3 $$\frac{{S_{y}^{4} }}{{D_{k} }}\left( \begin{gathered} \left( \begin{gathered} V_{04,k}^{6} + \left( {25V_{40}^{2} - 10V_{22,k}^{2} } \right)V_{04,k}^{4} + V_{04,k}^{2} \left( {8V_{22,k}^{{}} - 40V_{22,k}^{{}} V_{40}^{2} } \right) \hfill \\ + 16V_{22,k}^{2} V_{40}^{2} \hfill \\ \end{gathered} \right) \hfill \\ \,\,\,\,\,\,\,\,\,\,\,\,\,\,\,\,\,\,\,\,\,\,\,\,\,\,\,\,\,\,\,\,\,\,\, - 16V_{04,k}^{2} V_{40}^{2} + 16V_{22,k}^{2} - D_{k} V_{40} \left( {1 - \frac{{V_{22}^{2} }}{{V_{40} V_{04} }}} \right) \hfill \\ \end{gathered} \right) < 0.$$

**Condition 5.** By using (37), (38), (39) and (12) we can write

$$MSE\left( {\tau_{Pi} } \right) - MSE\left( {\tau_{5} } \right) < 0$$, i = 1,2,3.

For i = 1 $$\frac{{S_{y}^{4} }}{{A_{3,k}^{{}} }}\left[ {\frac{1}{16}\left\{ \begin{gathered} 16V_{22,k}^{{}} \left\{ {V_{22,k}^{{}} \left( {4V_{40}^{{}} - 3V_{04,k}^{{}} + 4} \right) + \left( {3V_{04,k}^{2} - 8V_{40}^{{}} V_{04,k}^{{}} } \right)} \right\} + \hfill \\ 9V_{04,k}^{3} + 64V_{04,k}^{{}} \left( {V_{04,k}^{{}} - 1} \right)V_{40}^{{}} \hfill \\ \hfill \\ - 16A_{3,k}^{{}} \left( \begin{subarray}{l} \left\{ {1 - \frac{1}{4}q^{2} \left( {1 + \theta } \right)^{2} V_{04} } \right\} - \\ \frac{{\left\{ {1 - \tfrac{1}{8}q^{2} \left( {1 + 3\theta + 4\theta^{2} } \right)V_{04} } \right\}^{2} }}{{1 - \frac{1}{4}q^{2} \theta \left( {1 + 3\theta } \right)V_{04}^{2} + V_{40} \left( {1 - \frac{{V_{22}^{2} }}{{V_{04} V_{40} }}} \right)}} \end{subarray} \right) \hfill \\ \end{gathered} \right\}} \right] < 0$$.

For i = 2 $$\frac{{S_{y}^{4} }}{D}\left( \begin{gathered} \left( \begin{gathered} V_{04}^{6} + \left( {25V_{40}^{2} - 10V_{22}^{2} } \right)V_{04}^{4} + \hfill \\ V_{04}^{2} \left( {8V_{22}^{{}} - 40V_{22}^{{}} V_{40}^{2} } \right) + 16V_{22}^{2} V_{40}^{2} \hfill \\ \end{gathered} \right) - 16V_{04}^{2} V_{40}^{2} + 16V_{22}^{2} - \hfill \\ D\left( {\left\{ {1 - \frac{1}{4}q^{2} \left( {1 + \theta } \right)^{2} V_{04} } \right\} - \frac{{\left\{ {1 - \tfrac{1}{8}q^{2} \left( {1 + 3\theta + 4\theta^{2} } \right)V_{04} } \right\}^{2} }}{{1 - \frac{1}{4}q^{2} \theta \left( {1 + 3\theta } \right)V_{04}^{2} + V_{40} \left( {1 - \frac{{V_{22}^{2} }}{{V_{04} V_{40} }}} \right)}}} \right) \hfill \\ \end{gathered} \right) < 0$$.

For i = 3 $$\frac{{S_{y}^{4} }}{{D_{k} }}\left( \begin{gathered} \left( \begin{gathered} V_{04,k}^{6} + \left( {25V_{40}^{2} - 10V_{22,k}^{2} } \right)V_{04,k}^{4} + \hfill \\ V_{04,k}^{2} \left( {8V_{22,k}^{{}} - 40V_{22,k}^{{}} V_{40}^{2} } \right) + 16V_{22,k}^{2} V_{40}^{2} \hfill \\ \end{gathered} \right) - 16V_{04,k}^{2} V_{40}^{2} + 16V_{22,k}^{2} \hfill \\ - D_{k} \left\{ {1 - \frac{1}{4}q^{2} \left( {1 + \theta } \right)^{2} V_{04} } \right\} - \frac{{\left\{ {1 - \tfrac{1}{8}q^{2} \left( {1 + 3\theta + 4\theta^{2} } \right)V_{04} } \right\}^{2} }}{{1 - \frac{1}{4}q^{2} \theta \left( {1 + 3\theta } \right)V_{04}^{2} + V_{40} \left( {1 - \frac{{V_{22}^{2} }}{{V_{04} V_{40} }}} \right)}} \hfill \\ \end{gathered} \right) < 0.$$

**Condition 6.** By using Eqs. (37), (38), (39) and (14) we can write

$$MSE\left( {\tau_{Pi} } \right) - MSE\left( {\tau_{6} } \right) < 0$$, i = 1,2,3.

For i = 1. $$\begin{gathered} 16V_{22,k}^{{}} \left\{ {V_{22,k}^{{}} \left( {4V_{40}^{{}} - 3V_{04,k}^{{}} + 4} \right) + \left( {3V_{04,k}^{2} - 8V_{40}^{{}} V_{04,k}^{{}} } \right)} \right\} + \hfill \\ 9V_{04,k}^{3} + 64V_{04,k}^{{}} \left( {V_{04,k}^{{}} - 1} \right)V_{40}^{{}} - \hfill \\ A_{3,k}^{{}} \left( {16MSE\left( {\tau_{4} } \right) - \frac{{\left[ {\left( {V_{40}^{{}} - 1} \right) + 8V_{04}^{{}} \left( {1 - \frac{{V_{22}^{2} }}{{V_{40}^{{}} V_{04}^{{}} }}} \right)} \right]}}{{4\left\{ {1 + \left\{ {V_{40}^{{}} - \frac{{V_{22}^{2} }}{{V_{04}^{{}} }}} \right\}} \right\}}}} \right) < 0 \hfill \\ \end{gathered}$$.

For i = 2.$$\begin{gathered} \left( \begin{gathered} V_{04}^{6} + \left( {25V_{40}^{2} - 10V_{22}^{2} } \right)V_{04}^{4} + \hfill \\ V_{04}^{2} \left( {8V_{22}^{{}} - 40V_{22}^{{}} V_{40}^{2} } \right) + 16V_{22}^{2} V_{40}^{2} \hfill \\ \end{gathered} \right) - 16V_{04}^{2} V_{40}^{2} + 16V_{22}^{2} - \hfill \\ D\left( {MSE\left( {\tau_{4} } \right) - \frac{{\left[ {\left( {V_{40}^{{}} - 1} \right) + 8V_{04}^{{}} \left( {1 - \frac{{V_{22}^{2} }}{{V_{40}^{{}} V_{04}^{{}} }}} \right)} \right]}}{{64\left\{ {1 + \left\{ {V_{40}^{{}} - \frac{{V_{22}^{2} }}{{V_{04}^{{}} }}} \right\}} \right\}}}} \right) < 0 \hfill \\ \end{gathered}$$.

For i = 3 $$\begin{gathered} \left( \begin{gathered} V_{04,k}^{6} + \left( {25V_{40}^{2} - 10V_{22,k}^{2} } \right)V_{04,k}^{4} + \hfill \\ V_{04,k}^{2} \left( {8V_{22,k}^{{}} - 40V_{22,k}^{{}} V_{40}^{2} } \right) + 16V_{22,k}^{2} V_{40}^{2} \hfill \\ \end{gathered} \right) - 16V_{04,k}^{2} V_{40}^{2} + 16V_{22,k}^{2} \hfill \\ - D_{k} \left( {MSE\left( {\tau_{4} } \right) - \frac{{\left[ {\left( {V_{40}^{{}} - 1} \right) + 8V_{04}^{{}} \left( {1 - \frac{{V_{22}^{2} }}{{V_{40}^{{}} V_{04}^{{}} }}} \right)} \right]}}{{64\left\{ {1 + \left\{ {V_{40}^{{}} - \frac{{V_{22}^{2} }}{{V_{04}^{{}} }}} \right\}} \right\}}}} \right) < 0. \hfill \\ \end{gathered}$$.

**Condition 7.** By using (37), (38), (39) and (16) we can write

$$MSE\left( {\tau_{Pi} } \right) - MSE\left( {\tau_{7} } \right) < 0$$, i = 1,2,3.

For i = 1. $$\begin{gathered} 16V_{22,k}^{{}} \left\{ {V_{22,k}^{{}} \left( {4V_{40}^{{}} - 3V_{04,k}^{{}} + 4} \right) + \left( {3V_{04,k}^{2} - 8V_{40}^{{}} V_{04,k}^{{}} } \right)} \right\} + \hfill \\ 9V_{04,k}^{3} + 64V_{04,k}^{{}} \left( {V_{04,k}^{{}} - 1} \right)V_{40}^{{}} \hfill \\ - \frac{{A_{3,k}^{{}} }}{{A_{3}^{{}} }}\left[ {\left\{ \begin{gathered} 16V_{22}^{{}} \left\{ {V_{22}^{{}} \left( {4V_{40}^{{}} - 3V_{04}^{{}} + 4} \right) + \left( {3V_{04}^{2} - 8V_{40}^{{}} V_{04}^{{}} } \right)} \right\} \hfill \\ \,\,\,\,\,\,\,\,\,\,\,\,\,\,\,\,\,\,\,\,\,\,\,\,\,\,\,\,\,\,\,\,\,\,\,\,\,\,\,\, + 9V_{04}^{3} + 64V_{04}^{{}} \left( {V_{04}^{{}} - 1} \right)V_{40}^{{}} \hfill \\ \end{gathered} \right\}} \right] < 0 \hfill \\ \end{gathered}$$.

For i = 2.$$\begin{gathered} \left( \begin{gathered} V_{04}^{6} + \left( {25V_{40}^{2} - 10V_{22}^{2} } \right)V_{04}^{4} + \hfill \\ V_{04}^{2} \left( {8V_{22}^{{}} - 40V_{22}^{{}} V_{40}^{2} } \right) + 16V_{22}^{2} V_{40}^{2} \hfill \\ \end{gathered} \right) - 16V_{04}^{2} V_{40}^{2} + 16V_{22}^{2} - \hfill \\ \frac{D}{{16A_{3}^{{}} }}\left[ {\left\{ \begin{gathered} 16V_{22}^{{}} \left\{ {V_{22}^{{}} \left( {4V_{40}^{{}} - 3V_{04}^{{}} + 4} \right) + \left( {3V_{04}^{2} - 8V_{40}^{{}} V_{04}^{{}} } \right)} \right\} \hfill \\ \,\,\,\,\,\,\,\,\,\,\,\,\,\,\,\,\,\,\,\,\,\,\,\,\,\,\,\,\,\,\,\,\,\,\,\,\,\,\,\, + 9V_{04}^{3} + 64V_{04}^{{}} \left( {V_{04}^{{}} - 1} \right)V_{40}^{{}} \hfill \\ \end{gathered} \right\}} \right] < 0 \hfill \\ \end{gathered}$$.

For i = 3. $$\begin{gathered} \left( \begin{gathered} V_{04,k}^{6} + \left( {25V_{40}^{2} - 10V_{22,k}^{2} } \right)V_{04,k}^{4} + \hfill \\ V_{04,k}^{2} \left( {8V_{22,k}^{{}} - 40V_{22,k}^{{}} V_{40}^{2} } \right) + 16V_{22,k}^{2} V_{40}^{2} \hfill \\ \end{gathered} \right) - 16V_{04,k}^{2} V_{40}^{2} + 16V_{22,k}^{2} \hfill \\ - \frac{{D_{k} }}{{16A_{3}^{{}} }}\left[ {\left\{ \begin{gathered} 16V_{22}^{{}} \left\{ {V_{22}^{{}} \left( {4V_{40}^{{}} - 3V_{04}^{{}} + 4} \right) + \left( {3V_{04}^{2} - 8V_{40}^{{}} V_{04}^{{}} } \right)} \right\} \hfill \\ \,\,\,\,\,\,\,\,\,\,\,\,\,\,\,\,\,\,\,\,\,\,\,\,\,\,\,\,\,\,\,\,\,\,\,\,\,\,\,\, + 9V_{04}^{3} + 64V_{04}^{{}} \left( {V_{04}^{{}} - 1} \right)V_{40}^{{}} \hfill \\ \end{gathered} \right\}} \right] < 0. \hfill \\ \end{gathered}$$.

**Condition 8.** By using (37), (38), (39) and (19), we can write

$$MSE\left( {\tau_{Pi} } \right) - MSE\left( {\tau_{8} } \right) < 0$$, i = 1,2,3.

For i = 1. $$   \begin{gathered}   16V_{{22,k}}^{{}} \left\{ {V_{{22,k}}^{{}} \left( {4V_{{40}}^{{}}  - 3V_{{04,k}}^{{}}  + 4} \right) + \left( {3V_{{04,k}}^{2}  - 8V_{{40}}^{{}} V_{{04,k}}^{{}} } \right)} \right\} \hfill \\    + 9V_{{04,k}}^{3}  + 64V_{{04,k}}^{{}} \left( {V_{{04,k}}^{{}}  - 1} \right)V_{{40}}^{{}}  \hfill \\    - A_{{3,k}}^{{}} \left\{ {\frac{{MSE\left( {\tau _{4} } \right)}}{{S_{y}^{4} }} - \frac{{\left\{ {\left( {V_{{40}}^{{}}  - \frac{{V_{{22}} ^{2} }}{{V_{{04}}^{{}} }}} \right) + \frac{1}{4}V_{{04}} ^{2} } \right\}}}{{\left\{ {1 + \left( {V_{{40}}^{{}}  - \frac{{V_{{22}} ^{2} }}{{V_{{04}}^{{}} }}} \right)} \right\}}}} \right\} < 0 \hfill \\  \end{gathered}   $$.

For i = 2.$$   \begin{gathered}   \left( \begin{gathered}   V_{{04}}^{6}  + \left( {25V_{{40}}^{2}  - 10V_{{22}}^{2} } \right)V_{{04}}^{4}  +  \hfill \\   V_{{04}}^{2} \left( {8V_{{22}}^{{}}  - 40V_{{22}}^{{}} V_{{40}}^{2} } \right) + 16V_{{22}}^{2} V_{{40}}^{2}  \hfill \\  \end{gathered}  \right) - 16V_{{04}}^{2} V_{{40}}^{2}  + 16V_{{22}}^{2}  \hfill \\   \,\,\,\,\,\,\,\,\,\,\,\,\,\,\,\,\,\, - D\left\{ {\frac{{MSE\left( {\tau _{4} } \right)}}{{S_{y}^{4} }} - \frac{{\left\{ {\left( {V_{{40}}^{{}}  - \frac{{V_{{22}} ^{2} }}{{V_{{04}}^{{}} }}} \right) + \frac{1}{4}V_{{04}} ^{2} } \right\}}}{{\left\{ {1 + \left( {V_{{40}}^{{}}  - \frac{{V_{{22}} ^{2} }}{{V_{{04}}^{{}} }}} \right)} \right\}}}} \right\} < 0 \hfill \\  \end{gathered}  $$.

For i = 3 $$    \begin{gathered}   \left( \begin{gathered}   V_{{04,k}}^{6}  + \left( {25V_{{40}}^{2}  - 10V_{{22,k}}^{2} } \right)V_{{04,k}}^{4}  +  \hfill \\   V_{{04,k}}^{2} \left( {8V_{{22,k}}^{{}}  - 40V_{{22,k}}^{{}} V_{{40}}^{2} } \right) + 16V_{{22,k}}^{2} V_{{40}}^{2}  \hfill \\  \end{gathered}  \right) - 16V_{{04,k}}^{2} V_{{40}}^{2}  + 16V_{{22,k}}^{2}  \hfill \\    - D_{k} \left\{ {\frac{{MSE\left( {\tau _{4} } \right)}}{{S_{y}^{4} }} - \frac{{\left\{ {\left( {V_{{40}}^{{}}  - \frac{{V_{{22}} ^{2} }}{{V_{{04}}^{{}} }}} \right) + \frac{1}{4}V_{{04}} ^{2} } \right\}}}{{\left\{ {1 + \left( {V_{{40}}^{{}}  - \frac{{V_{{22}} ^{2} }}{{V_{{04}}^{{}} }}} \right)} \right\}}}} \right\} < 0. \hfill \\  \end{gathered}   $$.

**Condition 9.** By using (37), (38), (39) and (20), we can write

$$MSE\left( {\tau_{Pi} } \right) - MSE\left( {\tau_{9} } \right) < 0$$, i = 1,2,3.

For i = 1 $$\frac{{S_{y}^{4} }}{{A_{3,k}^{{}} }}\left[ {\left\{ \begin{gathered} 16V_{22,k}^{{}} \left\{ {V_{22,k}^{{}} \left( {4V_{40}^{{}} - 3V_{04,k}^{{}} + 4} \right) + \left( {3V_{04,k}^{2} - 8V_{40}^{{}} V_{04,k}^{{}} } \right)} \right\} + \hfill \\ 9V_{04,k}^{3} + 64V_{04,k}^{{}} \left( {V_{04,k}^{{}} - 1} \right)V_{40}^{{}} \hfill \\ \hfill \\ - A_{3,k}^{{}} \left[ {\left\{ {V_{40}^{{}} + \Omega^{{}} \left( {\Omega V_{04}^{{}} + 2V_{22}^{{}} } \right)} \right\} - \frac{{D_{1}^{{}} D_{5}^{2} + D_{2}^{{}} D_{4}^{2} - 2D_{3}^{{}} D_{4}^{{}} D_{5}^{{}} }}{{D_{1}^{{}} D_{2}^{{}} - D_{3}^{2} }}} \right] \hfill \\ \end{gathered} \right\}} \right] < 0$$.

For i = 2 $$\frac{{S_{y}^{4} }}{D}\left( \begin{gathered} \left( \begin{gathered} V_{04}^{6} + \left( {25V_{40}^{2} - 10V_{22}^{2} } \right)V_{04}^{4} + \hfill \\ V_{04}^{2} \left( {8V_{22}^{{}} - 40V_{22}^{{}} V_{40}^{2} } \right) + 16V_{22}^{2} V_{40}^{2} \hfill \\ \end{gathered} \right) - 16V_{04}^{2} V_{40}^{2} + 16V_{22}^{2} \hfill \\ \,\, - D\left[ {\left\{ {V_{40}^{{}} + \Omega^{{}} \left( {\Omega V_{04}^{{}} + 2V_{22}^{{}} } \right)} \right\} - \frac{{D_{1}^{{}} D_{5}^{2} + D_{2}^{{}} D_{4}^{2} - 2D_{3}^{{}} D_{4}^{{}} D_{5}^{{}} }}{{D_{1}^{{}} D_{2}^{{}} - D_{3}^{2} }}} \right] \hfill \\ \end{gathered} \right) < 0$$.

For i = 3 $$\frac{{S_{y}^{4} }}{{D_{k} }}\left( \begin{gathered} \left( \begin{gathered} V_{04,k}^{6} + \left( {25V_{40}^{2} - 10V_{22,k}^{2} } \right)V_{04,k}^{4} + \hfill \\ V_{04,k}^{2} \left( {8V_{22,k}^{{}} - 40V_{22,k}^{{}} V_{40}^{2} } \right) + 16V_{22,k}^{2} V_{40}^{2} \hfill \\ \end{gathered} \right) - 16V_{04,k}^{2} V_{40}^{2} + 16V_{22,k}^{2} \hfill \\ - D_{k} \left[ {\left\{ {V_{40}^{{}} + \Omega^{{}} \left( {\Omega V_{04}^{{}} + 2V_{22}^{{}} } \right)} \right\} - \frac{{D_{1}^{{}} D_{5}^{2} + D_{2}^{{}} D_{4}^{2} - 2D_{3}^{{}} D_{4}^{{}} D_{5}^{{}} }}{{D_{1}^{{}} D_{2}^{{}} - D_{3}^{2} }}} \right] \hfill \\ \end{gathered} \right) < 0.$$

**Condition 10.** By using (37), (38), (39) and (24), we can write

$$MSE\left( {\tau_{Pi} } \right) - MSE\left( {\tau_{10} } \right) < 0$$, i = 1,2,3.

For i = 1 $$\frac{{S_{y}^{4} }}{{A_{3,k}^{{}} }}\left[ {\left\{ \begin{gathered} 16V_{22,k}^{{}} \left\{ {V_{22,k}^{{}} \left( {4V_{40}^{{}} - 3V_{04,k}^{{}} + 4} \right) + \left( {3V_{04,k}^{2} - 8V_{40}^{{}} V_{04,k}^{{}} } \right)} \right\} + \hfill \\ 9V_{04,k}^{3} + 64V_{04,k}^{{}} \left( {V_{04,k}^{{}} - 1} \right)V_{40}^{{}} \hfill \\ \hfill \\ - A_{3,k}^{{}} \left[ {1 - \frac{{\left[ {\left\{ {f_{3} \left( {0,2} \right)} \right\}^{2} - 2f_{2} \left( {0,2} \right)V_{40} + \left( {V_{04} - 1} \right)\left\{ {f_{2} \left( {0,2} \right)} \right\}^{2} } \right]}}{{\left( {V_{04} - V_{40}^{2} - 1} \right)}}} \right] \hfill \\ \end{gathered} \right\}} \right] < 0$$.

For i = 2 $$\frac{{S_{y}^{4} }}{D}\left( \begin{gathered} \left( \begin{gathered} V_{04}^{6} + \left( {25V_{40}^{2} - 10V_{22}^{2} } \right)V_{04}^{4} + \hfill \\ V_{04}^{2} \left( {8V_{22}^{{}} - 40V_{22}^{{}} V_{40}^{2} } \right) + 16V_{22}^{2} V_{40}^{2} \hfill \\ \end{gathered} \right) - 16V_{04}^{2} V_{40}^{2} + 16V_{22}^{2} \hfill \\ \,\, - D\left[ {1 - \frac{{\left[ {\left\{ {f_{3} \left( {0,2} \right)} \right\}^{2} - 2f_{2} \left( {0,2} \right)V_{40} + \left( {V_{04} - 1} \right)\left\{ {f_{2} \left( {0,2} \right)} \right\}^{2} } \right]}}{{\left( {V_{04} - V_{40}^{2} - 1} \right)}}} \right] \hfill \\ \end{gathered} \right) < 0$$.

For i = 3 $$\frac{{S_{y}^{4} }}{{D_{k} }}\left( \begin{gathered} \left( \begin{gathered} V_{04,k}^{6} + \left( {25V_{40}^{2} - 10V_{22,k}^{2} } \right)V_{04,k}^{4} + \hfill \\ V_{04,k}^{2} \left( {8V_{22,k}^{{}} - 40V_{22,k}^{{}} V_{40}^{2} } \right) + 16V_{22,k}^{2} V_{40}^{2} \hfill \\ \end{gathered} \right) - 16V_{04,k}^{2} V_{40}^{2} + 16V_{22,k}^{2} \hfill \\ - D_{k} \left[ {1 - \frac{{\left[ {\left\{ {f_{3} \left( {0,2} \right)} \right\}^{2} - 2f_{2} \left( {0,2} \right)V_{40} + \left( {V_{04} - 1} \right)\left\{ {f_{2} \left( {0,2} \right)} \right\}^{2} } \right]}}{{\left( {V_{04} - V_{40}^{2} - 1} \right)}}} \right] \hfill \\ \end{gathered} \right) < 0.$$

The condition mentioned above always hold true for all type of real data where the correlation is positive between the main study variable and supplementary variable.

## Empirical analysis

This section aims to investigate the performance of the proposed estimators against the competing estimators using data from some real-life situations. Table 1 consists of summary statistics of various datasets.

**Table 1:** Summary statistics of various data sets.

Summary statistics	Population I: Source^26^ Y: Amount (tons) of recyclable waste collection in Italy in 2003 X:Number of inhabitants in 2003	Population II: Source^23^ Y: The leaf area for the newly developed strain of wheat X: Weight of leaves	Population III: Source^27^ Y: weekly expenditure of food X: size of persons	Population IV: Source^28^ Y: Total amount of recyclable-waste collection in Italy (2003) X: Total amount of recyclable-waste collection in Italy (2002)
${\overline{\text{Y}}}$	51.82	26.84	27.49091	62.62
$\overline{X}$	11.26	106.20	3.7272	556.55
N	80	39	33	103
n	20	14	9	40
$S_{y}^{{}}$	18.35	6.24	9.976094	91.35
$S_{x}$	8.45	11.14	1.500198	610.16
$\phi_{40}$	2.26	2.26	5.72	37.12
$\phi_{04}$	2.86	2.99	2.380	17.87
$\phi_{22}$	2.22	2.40	1.43	17.22
$\rho_{yx}$	0.94	0.93	0.4237	0.72

The percentage relative efficiency (PRE) of all estimators discussed in the literature against $\tau_{1}$ has been used as performance index. The formula of PRE is given below

67 $$ PRE = \frac{{Var\left( {\tau_{1} } \right)}}{{MSE\left( {\tau_{i} } \right)\,or\,MSE\left( {\tau_{Pj,k} } \right)\,}} \times 100 $$

where, $i = 2,3, \cdots ,10$. J = 1,2,3. and k = 1,2 $\cdots$,6. are denoted by existing estimators and proposed estimators.

It is obvious from the Table 2 that the newly transformed estimators always perform well than existing estimators for all real data sets. The transformation introduced results high gain in efficiency. The first proposed estimator given by (25) is more efficient than the parent estimator suggested by^24^, moreover it also outperforms all the competing estimators discussed in the literature. The second proposed estimator given by (26) is more efficient even than our first proposed estimators and all other competing estimators. Further, incorporating the transformed auxiliary variable in the second proposed estimator generates the third proposed estimators given by (27) which are more efficient than all other estimators.

**Table 2:** PRE of proposed and competing estimators against the usual estimator $\tau_{1}^{{}}$.

Estimator		Population no.			
		1	2	3	4
Conventional estimators	$\tau_{1}^{{}}$	100.000	100.000	100.000	100.000
	$\tau_{2}^{{}}$	185.2941	280.000	102.4253	175.7390
	$\tau_{3}^{{}}$	249.5049	352.4475	101.1806	149.7570
	$\tau_{4}^{{}}$	274.0411	458.0562	102.9216	175.9545
	$\tau_{5}^{{}}$	195.2023	290.7025	124.2060	183.5658
	$\tau_{6}^{{}}$	289.34199	473.2631	153.7084	257.5825
	$\tau_{7}^{{}}$	336.50648	663.02559	159.1806	178.7570
	$\tau_{8}^{{}}$	302.428041	584.1095	169.9411	196.2725
	$\tau_{9}^{{}}$	236.64728	203.75595	102.7552	115.8661
	$\tau_{10}^{{}}$	269.8926	430.5612	101.5689	171.9047
Proposed estimators	$\tau_{P11}^{{}}$	339.83799	671.81178	171.4423	278.0721
	$\tau_{P13}^{{}}$	339.60901	664.96302	174.8473	279.5321
	$\tau_{P16}^{{}}$	341.674241	674.76219	169.2539	296.2645
	$\tau_{P2}^{{}}$	455.9280	1312.5474	218.2653	4059.0256
	$\tau_{P32}^{{}}$	456.2014	1421.83896	220.6593	4066.6241
	$\tau_{P34}^{{}}$	457.8603	1340.99191	216.2530	4080.5010
	$\tau_{P35}^{{}}$	278.8679	1314.1116	245.8746	4075.9032

## Simulation study

The simulation study of the suggested and existing estimators is conducted to assess the performance of both suggested and existing estimators. Three different populations of size 10,000 have been generated using positive correlation between main study and auxiliary variables. The intensity of correlation between the main study and the supplementary variable is high, moderate, and low in the first, the second and the third population respectively.

Population 1 $$\mu = \left[ {\begin{array}{*{20}c} {5.0} & {10.0} \\ \end{array} } \right]$$ $$\sum { = \left[ {\begin{array}{*{20}c} {9.0} & {2.9} \\ {2.9} & {1.0} \\ \end{array} } \right]}$$$$\rho = 0.9665$$.

Population 2 $$\mu = \left[ {\begin{array}{*{20}c} {5.0} & {10.0} \\ \end{array} } \right]$$ $$\sum { = \left[ {\begin{array}{*{20}c} {10.0} & {3.0} \\ {3.0} & {1.5} \\ \end{array} } \right]}$$$$\rho = 0.7559$$.

Population 3 $$\mu = \left[ {\begin{array}{*{20}c} {5.0} & {10.0} \\ \end{array} } \right]$$ $$\sum { = \left[ {\begin{array}{*{20}c} {10.0} & {1.5} \\ {1.5} & {1.5} \\ \end{array} } \right]}$$$$\rho = 0.3983$$.

We consider sample of sizes n = 50, 150, 300 are consider for each population, using simple random sampling without replacement approach. The steps below summarize the whole simulation procedure in R-Studio.

Step 1: Population is generated using Bivariate normal distribution with mean vector $$\mu = c\left( {\begin{array}{*{20}c} {5.0} & {10.0} \\ \end{array} } \right)$$ and $$\sum { = \left[ {\begin{array}{*{20}c} {9.0} & {2.9} \\ {2.9} & {1.0} \\ \end{array} } \right]}$$ and calculated all parameters of auxiliary variable and constant from the data.

Step 2: From each of the target population, samples of $n$ =  50, 150, 300 have been drawn using SRSWOR method respectively, allow the loop to 100,000 times, select the sample, and calculated the estimates in each iteration.

Step 3: Utilizing samples generated in step 2, the 100,000 values of $\tau_{i}^{{}} ,i = 1,2,..10.$ and $\tau_{Pjk} ,j = 1,2,3\,and\,k = 1,2,..,6.$, are obtained separately, using (4–20) and (23), (34) and (35), respectively.

Step 4: The estimates obtained from each iteration are store in a matrix and calculate the percent PRE averaging over all iteration of each estimates by using the formula (67), and report the results in Table 3.

**Table 3:** Simulation results for the percent relative efficiencies of the newly developed estimators and conventional estimators with respect to the usual estimator considering different sample sizes and correlation coefficients.

Estimators	Population								
	1			2			3		
	n = 50	n = 150	n = 300	n = 50	n = 150	n = 300	n = 50	n = 150	n = 300
$\tau_{1}^{{}}$	100.00	100.000	100.00	100.00	100.00	100.00	100.00	100.00	100.00
$\tau_{2}^{{}}$	195.36	198.9839	201.47	101.55	102.46	100.37	104.84	102.95	103.30
$\tau_{3}^{{}}$	93.38	97.6381	101.63	110.53	104.32	102.56	103.45	101.36	102.49
$\tau_{4}^{{}}$	212.54	209.3710	218.63	115.63	108.37	111.35	105.25	108.54	106.26
$\tau_{5}^{{}}$	223.58	201.2898	219.35	123.92	119.73	113.27	110.47	107.16	108.57
$\tau_{6}^{{}}$	235.64	208.7391	202.63	120.46	123.18	115.27	115.65	107.16	110.75
$\tau_{7}^{{}}$	238.29	230.4728	224.38	139.47	130.36	128.38	112.59	110.47	108.73
$\tau_{8}^{{}}$	235.73	202.3729	223.24	130.28	121.46	119.37	110.45	108.38	106.39
$\tau_{9}^{{}}$	232.27	228.4732	214.73	137.47	128.56	126.18	107.38	106.75	106.95
$\tau_{9}^{{}}$	198.76	203.2417	215.76	119.78	108.45	110.56	104.24	107.96	104.22
$\tau_{P1,1}^{{}}$	241.47	241.66	240.63	141.46	138.86	131.25	119.27	111.45	112.45
$\tau_{P1,4}^{{}}$	242.45	240.25	238.58	140.48	132.45	131.57	115.26	110.47	110.46
$\tau_{P1,6}^{{}}$	241.35	235.76	226.65	139.73	131.46	129.45	112.46	11.52	108.98
$\tau_{P2}^{{}}$	260.85	262.37	275.46	185.36	189.83	190.89	135.85	136.86	141.86
$\tau_{P3,1}^{{}}$	262.46	265.77	275.85	191.92	195.53	197.04	136.95	139.18	140.47
$\tau_{P3,3}^{{}}$	265.66	266.75	276.85	195.84	195.95	198.90	140.43	142.65	145.96
$\tau_{P3,5}^{{}}$	262.65	263.75	275.75	186.53	189.54	190.66	136.06	136.93	142.47
$\tau_{P3,6}^{{}}$	271.46	270.01	276.66	197.59	197.49	198.45	140.94	142.95	147.88

The simulation results indicate that the newly developed estimators are highly efficient as compared to the conventional estimators in all situations. It is obvious that for the high correlation $\rho \ge 0.96$, the estimators are tend to be more efficient than moderate $\rho \ge 0.75$ and low $\rho \ge 0.39$ correlation. Thus, the intensity of relationship between the study and supplementary variable plays a vital role in increasing the efficiency. The newly developed estimator retains its efficiency in all three cases. Further, the transformation also plays a remarkable role in enhancing efficiency. As it can be seen from the simulation in the Table 3, the first transformed proposed estimator is efficient than its parent estimator $\tau_{7}$ for different choices of $\gamma_{1\,\,} \,\,and\,\,\gamma_{2\,\,}$. Similarly, the third proposed estimator is also efficient not only from the conventional estimators but also from the second proposed estimator for different choices of $\gamma_{1\,\,} \,\,and\,\,\gamma_{2\,\,}$.

## Conclusion

The empirical and simulation findings highlight the remarkable efficiency of the newly developed estimators in comparison to conventional counterparts across diverse scenarios. Particularly noteworthy is the enhanced efficiency of these estimators in situations with higher correlation, emphasizing the pivotal role of the strength of the relationship between the study and supplementary variable. This aligns with the expectation that a stronger correlation contributes to increased estimator efficiency.

Furthermore, the robustness of the newly developed estimator is evident across all correlation levels—high, moderate, and low. This consistent performance underscores the versatility and reliability of the proposed estimators, making them applicable in a broad spectrum of scenarios.

The introduced transformation in the estimators emerges as a key contributor to enhanced efficiency. As depicted in Table 3, simulation results affirm that the first transformed proposed estimator consistently outperforms its parent estimator across various choices. Similarly, the third proposed estimator not only demonstrates efficiency over conventional estimators but also surpasses the second proposed estimator for different choices. This emphasizes the significant role played by transformations in elevating the overall performance of the estimators.

Additionally, the proposed estimators exhibit flexibility and effectiveness in various sampling designs, such as stratified random sampling, non-response sampling, and adaptive cluster sampling. The extension of these estimators to non-conventional sampling designs, including adaptive cluster sampling and stratified adaptive cluster sampling, is also under consideration for variance estimation. These estimators display flexibility in exploring potential improvements in formulating estimates of population parameter utilizing two auxiliary variables.

In conclusion, the proposed estimators shows remarkable efficiency in finite population variance estimation under the simple random sampling scheme without replacement. The encouraging findings suggest their applicability in diverse survey scenarios, and future research avenues could further enhance their adaptability, extending their utility to more intricate sampling designs.

## Data Availability

The datasets generated during and/or analyzed during the current study are available from the corresponding author on reasonable request.
